# The Third‐Generation Magnetic Super‐Stable Mineralizer: Complete Removal and Separation of Multiple Heavy Metal Pollutants

**DOI:** 10.1002/advs.75289

**Published:** 2026-04-15

**Authors:** Haoran Wang, Menghan Huang, Ruihua Mao, Xiaohan Zhang, Zhaohui Wu, Xiaofeng Pang, Tong Lin, Dongyuan Cui, Sai An, Yu‐Fei Song

**Affiliations:** ^1^ State Key Laboratory of Chemical Resource Engineering Beijing University of Chemical Technology Beijing P. R. China

**Keywords:** heavy metal, layered double hydroxide, magnetic separation, soil remediation, super‐stable mineralization

## Abstract

The retrieval of used mineralizers from multiple heavy metals contaminated environments is a critical challenge that remains to be solved regarding in situ super‐stable mineralization. Herein, the third‐generation magnetic mineralizer represented by the M‐MgAl‐700 was successfully fabricated with a typical core‐shell structure, strong superparamagnetism, a large specific surface area, and abundant oxygen defects. The resultant M‐MgAl‐700 exhibited maximum mineralization capacities of 1173.80 and 93.11 mg g^‒1^ for individual Cd(II) and As(V), respectively, which markedly surpassed the majority of mineralizers reported so far. Moreover, the M‐MgAl‐700 can reduce the concentrations of both Cd(II) and As(V), no matter in co‐polluted water or soil, to levels compliant with national standards (GB8978‐1996 and GB15618‐2018), highlighting its versatile utility. Detailed quasi in situ characterization and density functional theory (DFT) calculation collectively revealed the diverse removal mechanisms of topological transformation to form the CdAl‐CO_3_ for Cd(II) and surface adsorption via a binuclear bidentate mode for As(V), respectively. More crucially, the mineralized products with adequate magnetism allowed for highly efficient and thorough isolation from water and soil by using the magnet. This work presents the third‐generation magnetic super‐stable mineralizer that integrates high‐capacity mineralization with rapid, facile, and complete recoverability, offering a cross‐disciplinary platform for the development of advanced environmental remediation.

## Graphical abstract

The 3^rd^‐generation magnetic super‐stable mineralizer M‐MgAl‐700 features a core‐shell structure, strong superparamagnetism, large surface area, and abundant oxygen defects. It achieves exceptional Cd(II) and As(V) mineralization capacities, reducing both pollutants in co‐contaminated water and soil to meet national standards. The mineralized products enable complete magnetic recovery, which is in good contrast compared with previous mineralizers reported so far.

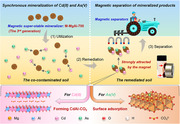

## 1 Introduction

The cadmium (Cd(II)) and arsenate (As(V)), prevalent toxic heavy metal cations and metalloid contaminants [1], are frequently introduced into aquatic and terrestrial environments through anthropogenic activities such as industrial electroplating, metal smelting, mining, and agricultural practice, resulting in their widespread co‐occurrence and combined pollution [2]. As Cd(II) and As(V) coexist, they may produce synergistic, additive, or antagonistic effects, significantly increasing ecological risks and the difficulty of governance [3]. This complex co‐contamination can damage soil functions, endanger the safety of crops, and pose threats to human health through the food chain, thus creating a pressing requirement for more advanced pollution control technologies. Different from the commonly used remediation techniques for pollutants of multiple heavy metals encompassing elution, soil replacement, and phytoremediation, in situ mineralization method holds great potential owing to its unique advantages, including high efficiency, strong operability, the ability for “menu‐style customization” of mineralizer, and low cost [4, 5].

**SCHEME 1:**
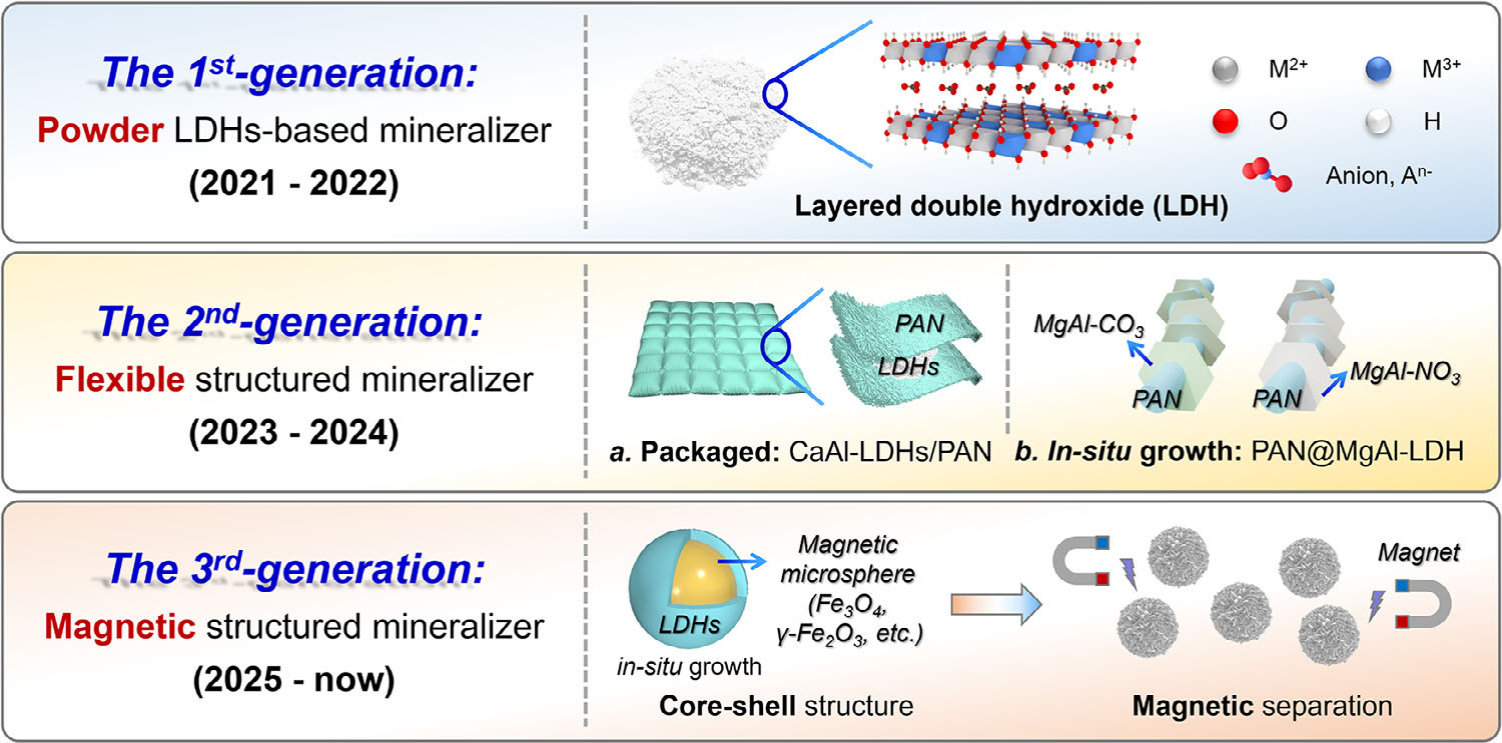
The schematic diagram of the development process of the super‐stable mineralizer for heavy metals.

Layered double hydroxides (abbreviated as LDHs) are a class of well‐recognized powder mineralizers for various heavy metals [6]. The presence of numerous interactions (e.g., electrostatic attraction, hydrogen bonding, covalent bonding, and intermolecular forces) in LDHs enable them to achieve: 1) super‐stable mineralization structure (SSMS) due to the extremely low *K_sp_* (10^‒40^‐10^‒60^) [7]; 2) synchronous removal of coexisting heavy metals via distinct mineralizing mechanisms, such as isomorphic substitution for Ni(II) and Cu(II) [8], precipitation for Pb(II) and Cd(II) [9], interlayer confinement for Cr(VI) [10], and surface adsorption for As(III/V) and Se(IV) [11], among others [12]; 3) reliable acid‐base resistance (4.0 < pH < 12.0) to balance the “trade‐off” effect arising from the different occurrence states and physical‐chemical properties of heavy metal cations and anions in specific pH values [13]. Therefore, LDHs‐based powders, as the first‐generation super‐stable mineralizer, have been widely applied in the treatment of multiple heavy metal contamination, achieving excellent results and presenting a very promising future (Scheme 1).

Later on, the practical challenges of separation difficulty, secondary pollution risk, and powder agglomeration and loss have motivated the development of a second‐generation flexible structured mineralizer, which was displayed in Scheme 1. In 2019, Song et al. prepared the novel PAN@LDH utilizing “seeds embedded epitaxial growth” (SEEG) strategy, in which these types of inorganic‐organic composite membranes can effectively immobilize the powdery LDHs, dispersed active sites, as well as circumvent powder separation issues [14]. Afterwards, the resultant PAN@LDHs with adjustable properties were increasingly deployed in the capture of multiple heavy metal and/or anions (e.g., Cu(II), Cr(VI), Se(IV), etc.), showing operational simplicity [15], separable recyclability [10], and reusability [16] that the first‐generation mineralizer did not possess. Additionally, based on the size matching considerations, a filter bag‐shaped LDHs/PAN mineralizer was fabricated, which can selectively retain the powdery LDHs while permitting the diffusion of heavy metal ions, realizing the separation of mineralization products [17]. It is found that the second‐generation mineralizer has made significant progress at the laboratory scale. Despite its functional promise, the composite membrane materials are facing major practical limitations, including the issues with efficiency of use, economic cost, and labor force, that present pressing technical bottlenecks and need to be immediately addressed.

Iron‐based magnetic nanoparticles (such as Fe_3_O_4_, *γ*‐Fe_2_O_3_, etc.) are the functional materials predominantly composed of the iron (Fe) element or its compound [18], featuring the nanoscale dimensions and distinct magnetic responsiveness [19]. Take the relatively common case of *γ*‐Fe_2_O_3_ as an example, the advantages lie in the combination of high saturation magnetization, good biocompatibility, environmental friendliness, and low cost, which facilitates them to demonstrate broad application prospects in the field of environmental remediation [20]. To combine the advantages of magnetic materials and LDHs, researchers construct a core‐shell composite via in situ growth of LDHs on the surface of a magnetic core [21], which is also the prototype of our third‐generation magnetic super‐stable mineralizer. By leveraging an external magnetic field, it can achieve rapid and thorough separation of post‐reaction mineralizers within seconds, with very simple operation and extremely low energy consumption [22]. However, versus pure LDHs, the composite mineralizers suffer from a performance compromise on a reduced proportion of effective components, which leads to inferior heavy metal removal [23]. This inherent “trade‐off” effect thus defines the central optimization problem, necessitating urgent management.

In this work, we report the invention of a novel type of third‐generation magnetic structured mineralizer by adopting the magnetic LDH precursors for high‐temperature calcination (Scheme 1). The appropriate temperature can retain sufficient magnetism of the mineralizer while removing the hydroxyl groups and interlayer anions from the laminates of LDHs, thereby causing a structural transformation to form the mixed metal oxides (MMOs). This approach can significantly enhance the mineralization capacities for various heavy metals, which demonstrates the most distinctive advantage of the third‐generation magnetic structured mineralizer. By regulating the metal elements (Mg(II), Zn(II), Al(III), and Fe(III), etc.), the expansion synthesis of such magnetic super‐stable mineralizers can be realized, verifying the universality of this synthetic strategy. The selected sample (denoted as M‐MgAl‐700) simultaneously exhibited efficient removal performance towards both Cd(II) and As(V) and the property of immediate and facile magnetic separation, achieving satisfactory results on both super‐stable mineralization and thorough separation no matter in water or soil remediation experiments. Detailed quasi in situ characterization, including X‐ray diffraction (XRD), X‐ray absorption fine structure (XAFS), and DFT calculation jointly revealed different removal mechanisms of topological transformation for Cd(II) and surface adsorption for As(V), respectively. This study offers unique insights into the technical challenges of accomplishing both excellent performance and complete separation in actual applications.

## 2 Results and Discussion

### 2.1 Morphological and Structural Characterization

**FIGURE 1:**
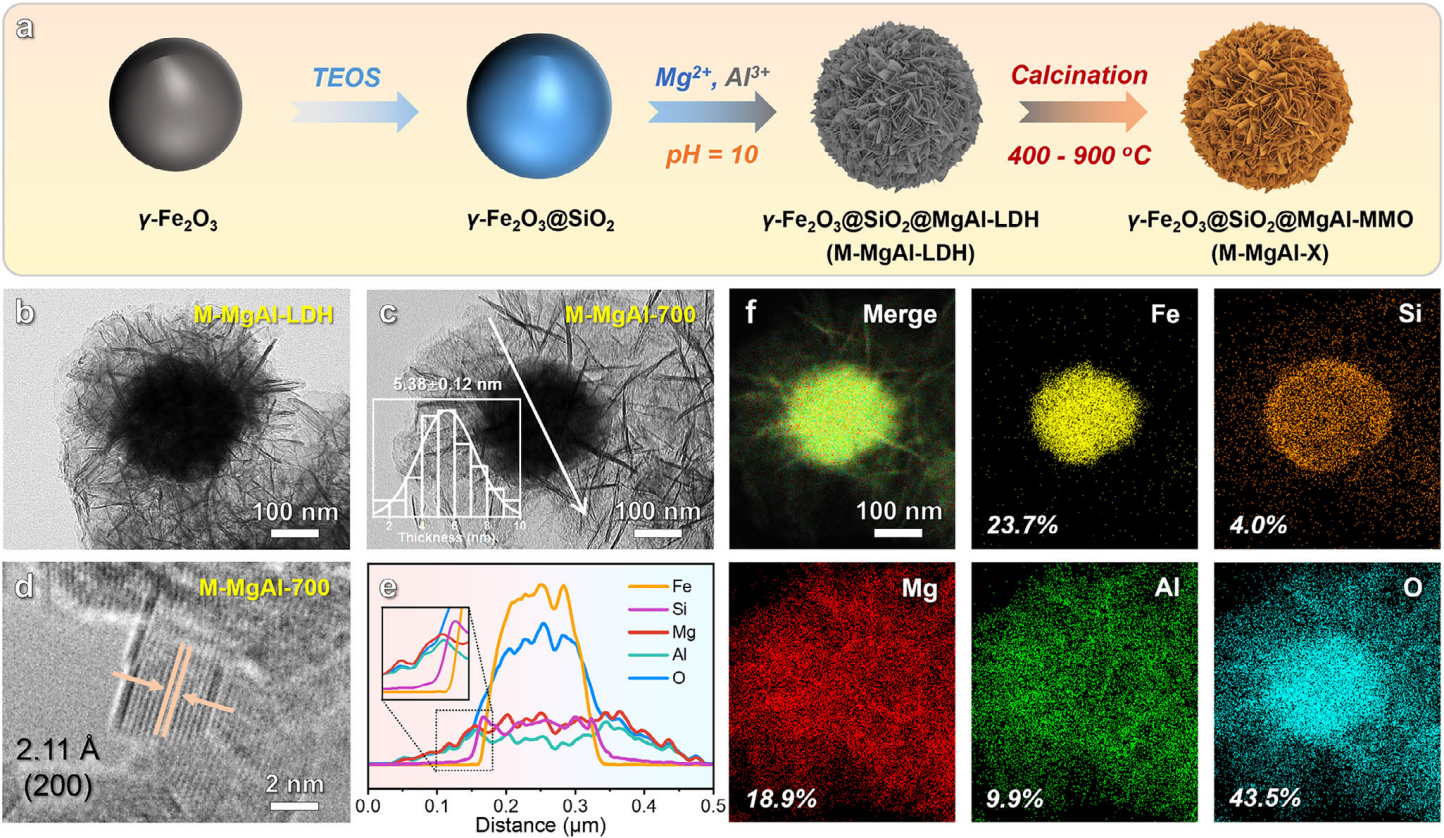
The synthetic route and morphological characterization of the M‐MgAl‐X. (a) The schematic diagram of the preparation process for M‐MgAl‐X; HRTEM images of (b) M‐MgAl‐LDH and (c–e) M‐MgAl‐700 (Inset: thickness distribution graph of the outer nanosheets); EDX (d) line scanning and (f) mapping data of M‐MgAl‐700.

As presented in Figure 1a, the successful synthesis of M‐MgAl‐X went through four steps: 1) Using the solvothermal method to obtain the *γ*‐Fe_2_O_3_ [24]; 2) Preparing the *γ*‐Fe_2_O_3_@SiO_2_ via the sol‐gel method [22]; 3) The in situ growth of MgAl‐LDH on the surface of *γ*‐Fe_2_O_3_@SiO_2_ by co‐precipitation method (the product was abbreviated as M‐MgAl‐LDH) [25]; 4) Calcining M‐MgAl‐LDH within the range of 400 to 900°C to form the M‐MgAl‐X [26]. The scanning electron microscopy (SEM) image in Figure S1a displayed that *γ*‐Fe_2_O_3_ presented a spherical morphology with a diameter of ca. 250 nm. After coating with SiO_2_, the size of the microspheres did not change, but the surface became much smoother than the pure *γ*‐Fe_2_O_3_ (Figure S1b). Moreover, in Figure 1b,c, high‐resolution transmission electron microscopy (HRTEM) revealed the typical core‐shell structure of both M‐MgAl‐LDH and M‐MgAl‐X (take M‐MgAl‐700 for example), i.e., there was no obvious change in the morphology of the resultant mineralizer before and after calcination. It was measured that the nanosheets with a thickness of ca. 5.38 nm were uniformly grown on the surface of microspheres. In addition, the nanosheets showed the lattice spacing of 1.96 and 2.11 Å in Figure S2 and Figure 1d, which can be attributed to the (018) crystal plane of MgAl‐LDH [27]. and the (200) plane of MgO phase [28], respectively, confirming the formation of the corresponding products.

The elemental distribution of M‐MgAl‐LDH, as well as M‐MgAl‐700 was also monitored. Take the M‐MgAl‐700 as an example, in Figure 1e, the order of the peak positions for each element obtained from energy‐dispersive X‐ray (EDX) line scanning data was as follows: Mg, Al, and O < Si < Fe, indicating that the outermost layer of the core‐shell structure predominantly comprised Mg, Al, and O elements, the middle layer was composed of Si element, and Fe element was distributed in the innermost core. Besides, the mapping data assisted in proving the homogeneous distribution of each element on the core or shell of M‐MgAl‐700, which was consistent with the line scanning data (Figure 1f). Moreover, it can be seen that the atomic ratio of Mg to Al was approximately 2:1, aligning with the feed ratio. The relevant data of M‐MgAl‐LDH were compatible with that of M‐MgAl‐700, which was shown in Figures S3 and S4. Similarly, after the sample was digested with strong acid, the concentrations of various ions in the solution were detected by Inductively Coupled Plasma Optical Emission Spectrometer (ICP‐OES). The calculated atomic ratio matched well with the mapping data (Table S1).

**FIGURE 2:**
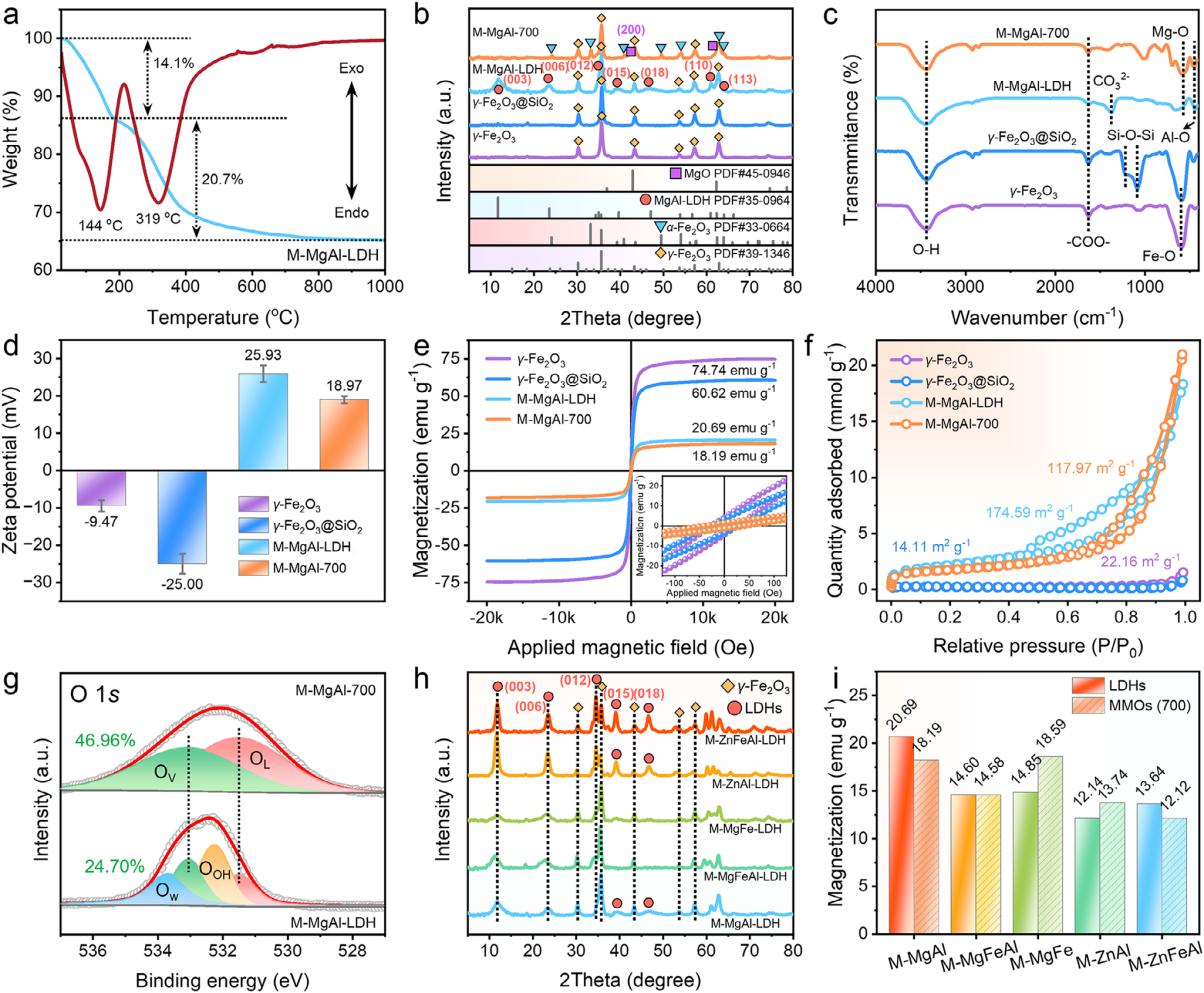
The characterization of structure and physical‐chemical properties for M‐MgAl‐X. (a) The TG curves of M‐MgAl‐LDH; (b) XRD patterns, (c) FT‐IR spectra, (d) zeta potential diagram, (e) room‐temperature magnetization hysteresis loops (Inset: magnified hysteresis loops), and (f) N_2_ adsorption‐desorption isotherms of *γ*‐Fe_2_O_3_, *γ*‐Fe_2_O_3_@SiO_2_, M‐MgAl‐LDH, and M‐MgAl‐700; (g) O 1*s* XPS spectra of M‐MgAl‐LDH and M‐MgAl‐700; (h) XRD patterns of various magnetic LDHs; (i) saturation magnetization values diagram of various magnetic LDHs and MMOs.

More detailed characterization about the structures and properties of the *γ*‐Fe_2_O_3_, *γ*‐Fe_2_O_3_@SiO_2_, M‐MgAl‐LDH, and M‐MgAl‐X was implemented. According to Figure 2a and Figure S5, the thermogravimetric‐differential thermal analysis (TG‐DTA) was firstly performed to detect the weight loss during the topotactic transformation process. It can be found that the synthesized *γ*‐Fe_2_O_3_ and *γ*‐Fe_2_O_3_@SiO_2_ presented great thermal stability, with the weight loss rate less than 10.0% below 800°C. The weight‐loss plateau of M‐MgAl‐LDH was similar to that of MgAl‐LDH, indicating the preservation of LDH structure [29]. Meanwhile, powder X‐ray diffraction (PXRD) analysis was conducted to determine the basic structure at each state of the synthetic process (Figure 2b; Figure S6). Obviously, both *γ*‐Fe_2_O_3_ and *γ*‐Fe_2_O_3_@SiO_2_ were in perfect agreement with the standard pattern (PDF#39‐1346), proving the effective synthesis of *γ*‐Fe_2_O_3_ and the coating of the amorphous SiO_2_. After growth of LDHs, M‐MgAl‐LDH simultaneously exhibited the characteristic peaks of both *γ*‐Fe_2_O_3_ and MgAl‐LDH, i.e., M‐MgAl‐LDH not only retained the typical peaks of *γ*‐Fe_2_O_3_, but also showed the representative (00*l*) and (110) basal reflection peaks (PDF#35‐0964), which validated the coexistence of two phases [22]. With the calcination temperature gradually increased, the diffraction peaks of MgAl‐LDHs first disappeared in 400°C and were replaced by those of MgO in 600°C, while no feature of Al_2_O_3_ can be detected, implying that the MgAl‐LDH part transformed to MgO (PDF#45‐0946) and amorphous Al_2_O_3_ phases. Besides, when the temperature exceeded 700°C, the diffraction peaks belonging to the *α*‐Fe_2_O_3_ (PDF#33‐0664) began to appear in calcined products, with the intensity progressively increasing, which suggested that a phase transition occurred in the Fe_2_O_3_ [30], giving rise to some of *γ*‐Fe_2_O_3_ converting into *α*‐Fe_2_O_3_. The above results also explained the macroscopic phenomenon of the color changing from black to brownish‐red in the powder (Figure S7).

Furthermore, Fourier transform infrared (FT‐IR) spectra and zeta potential tests were also carried out. As shown in Figure 2c and Figure S8, apart from the characteristic vibration peaks of Fe‒O at ∼590 cm^‒1^ observed in both *γ*‐Fe_2_O_3_ and *γ*‐Fe_2_O_3_@SiO_2_ [31], *γ*‐Fe_2_O_3_@SiO_2_ displayed additional peaks corresponding to the asymmetric stretching vibration of the Si‒O‒Si bond (1090 and 1220 cm^‒1^) [32]. The presence of CO_3_^2‒^ peaks at 1370 cm^‒1^ in M‐MgAl‐LDH, which vanished after being calcinated, served as evidence for its intercalation prior to structural collapse that led to its disappearance [33], while the peaks of Mg/Al‒O bonds remained present throughout. The surface charge of the composite evolved from negative to positive during the synthesis (Figure 2d; Figure S9). Starting at −9.47 mV for *γ*‐Fe_2_O_3_ owing to the existence of −OH groups on the surface and coordinated carboxylates from NaAC, it decreased to −25.00 mV after SiO_2_ coating due to the silanol groups [34]. A charge reversal to +25.93 mV occurred upon the in situ growth of LDHs: in alkali, SiO_2_ dissolved, and the exposed Si sites were bound to Mg(II) or Al(III) through the oxygen atoms, giving rise to the encapsulation by positively charged layers of LDHs [35]. The lower surface potential of M‐MgAl‐X, compared to its M‐MgAl‐LDH precursor, was primarily because of changes in surface chemical properties induced by calcination.

In Figure 2e, the magnetic characterization at room temperature with a vibrating sample magnetometer (VSM) showed that the saturation magnetization values of *γ*‐Fe_2_O_3_, *γ*‐Fe_2_O_3_@SiO_2_, M‐MgAl‐LDH, and M‐MgAl‐700 were 74.74, 60.62, 20.69, and 18.19 emu g^‒1^, respectively (see Figure S10 for other control samples). Due to the layer‐by‐layer coating of non‐magnetic substances (SiO_2_, MgAl‐LDH, MgO, and Al_2_O_3_) and phase transition from *γ*‐Fe_2_O_3_ to *α*‐Fe_2_O_3_ accompanied by the temperature increase, the values continued decreasing [36]. Regardless of which sample it was, the magnified hysteresis loops of each one confirmed its superparamagnetism [37]. As shown in Figure 2f and Figure S11, the porosity property of each sample was studied by the nitrogen adsorption‐desorption test. Since *γ*‐Fe_2_O_3_ possessed the inherent high density, and SiO_2_ was almost porous‐free [38], the Brunauer‐Emmett‐Teller (BET) specific surface areas of *γ*‐Fe_2_O_3_ and *γ*‐Fe_2_O_3_@SiO_2_ were both relatively low, being 22.16 and 14.11 m^2^ g^‒1^, respectively. The as‐prepared M‐MgAl‐LDH exhibited the BET surface areas of 174.59 m^2^ g^‒1^ and type IV adsorption‐desorption isotherms and type H2(b) hysteresis loops [39], suggesting their mesoporous structure owing to the stacking of MgAl‐LDH nanosheets. Notably, the specific surface area decreased especially after reaching the calcination temperature over 800°C (< 100.0 m^2^ g^‒1^), which can be attributed to the collapse of the layers, stacking, and high‐temperature sintering [40]. By performing the peak fitting on the O 1*s* spectrum of x‐ray photoelectron spectroscopy (XPS), the content of the oxygen defect in M‐MgAl‐LDH and M‐MgAl‐X can be quantitatively characterized (Figure 2g; Figure S12). As for M‐MgAl‐LDH, the total peak can be fitted into four deconvoluted peaks, which were ascribed to the lattice oxygen (O_L_), OH groups (O_OH_), oxygen vacancies (O_V_), and chemisorbed water (O_W_) at 531.5, 532.3, 533.1, and 533.7 eV, respectively [41]. However, in M‐MgAl‐X, the peaks of O_OH_ and O_W_ disappeared, caused by calcination, while the peak emergence positions of O_L_ and O_V_ remained constant. Compared with other samples, M‐MgAl‐700 displayed the highest content of O_V_ (46.96%), which was conducive to the removal of multiple heavy metals [9].

### 2.2 Expanding Synthesis of Various Magnetic Mineralizers

To verify the universality of the synthetic method, different metal elements were selected to prepare M‐MgFeAl‐LDH, M‐MgFe‐LDH, M‐ZnAl‐LDH, and M‐ZnFeAl‐LDH and their calcined derivatives (take X = 700 for instance). As shown in Figure 2h, the obtained magnetic LDHs concurrently featured the characteristic peaks belonging to both *γ*‐Fe_2_O_3_ and LDHs, supporting the success of extended synthesis. After calcination, all four types of magnetic LDHs underwent the topological transformations to the corresponding MMOs. For M‐MgFeAl‐700 and M‐MgFe‐700, in addition to the phase of *α*‐Fe_2_O_3_ and *γ*‐Fe_2_O_3_, the other peaks compared well with MgFe_2_O_4_ (PDF#17‐0464) [42], which was displayed in Figure S14a. In Zn‐based MMOs, the characteristic peaks of ZnO (PDF#36‐1451) were mainly observed (Figure S14b) [43]. The result of VSM tests in Figure 2i and Figure S15 demonstrated that various magnetic LDHs possessed the saturation magnetization value of approximately 15.0 emu g^‒1^, attesting to the strong superparamagnetism [37], which can be attracted and separated by a neodymium‐iron‐boron (NdFeB) magnet within an extremely short period of time (< 1 s). In brief, the in situ growth of distinct types of LDHs or MMOs on the surface of *γ*‐Fe_2_O_3_ magnetic microspheres can be achieved.

### 2.3 Removal Performance for Individual Cd(II) and as(V) in Aqueous Solution

**FIGURE 3:**
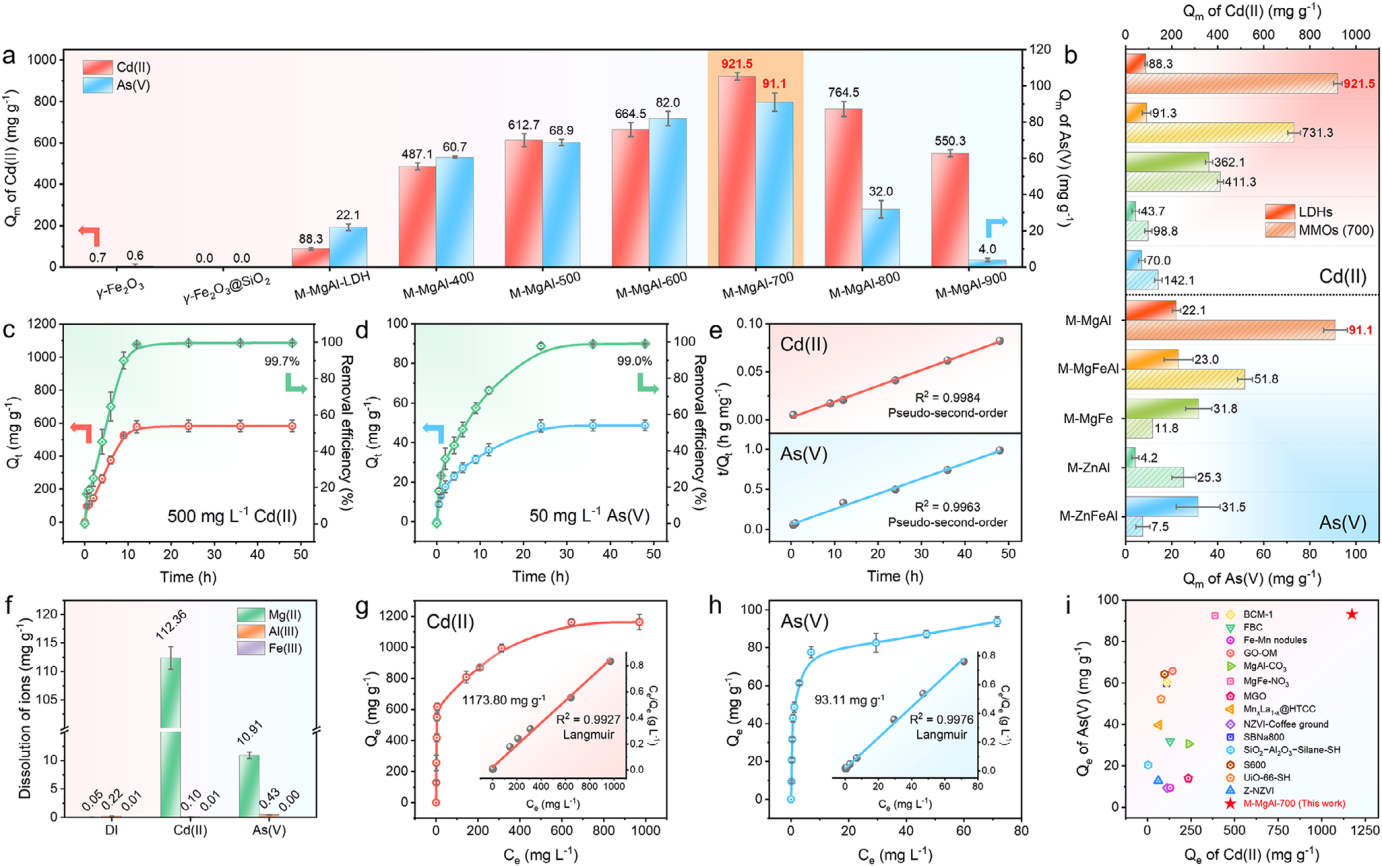
The removal performance of magnetic super‐stable mineralizers towards individual Cd(II) or As(V) in aqueous solution. The preliminary screening of mineralization performance by (a) the precursors (*γ*‐Fe_2_O_3_, *γ*‐Fe_2_O_3_@SiO_2_, and M‐MgAl‐LDH) and M‐MgAl‐X, and (b) various magnetic LDHs and MMOs; Removal performances and efficiencies of M‐MgAl‐700 in (c) Cd(II) and (d) As(V) solution with the different mineralization times; (e) Pseudo‐second‐order adsorption kinetics models fitted by linear regression; (f) Comparison of dissolution content of Mg(II), Al(III), and Fe(III) from M‐MgAl‐700 in DI, Cd(II), and As(V) solution; Removal performances of M‐MgAl‐700 in (g) Cd(II) and (h) As(V) solution with the concentration range from 10 to 2000 mg L^−1^ (Inset: Langmuir adsorption isotherms models fitted by linear regression); (i) Comparison of mineralizing performance for Cd(II) and As(V) by different mineralizers.

Firstly, the mineralization capacities for individual Cd(II) and As(V) of M‐MgAl‐X (X = 400–900) and the precursors (*γ*‐Fe_2_O_3_, *γ*‐Fe_2_O_3_@SiO_2_, and M‐MgAl‐LDH) were preliminarily investigated. As depicted in Figure 3a, with the calcination temperature increasing, the removal performance of the different M‐MgAl‐X presented a volcano‐like trend, which reached the peak when X = 700, with the value of 921.5 mg g^‒1^ for Cd(II) and 91.1 mg g^‒1^ for As(V), respectively. Since neither *γ*‐Fe_2_O_3_ nor *γ*‐Fe_2_O_3_ alone demonstrated the ability to capture Cd(II) and As(V), it can be inferred that the entire mineralization capacities were contributed by the component of MgAl‐LDH and its calcined derivatives. The analysis of O 1*s* XPS spectra in the previous text validated that oxygen vacancies enhanced mineralization performance, with M‐MgAl‐700 delivering the most superior activity (Figure 2g; Figure S12). Moreover, both M‐MgAl‐LDH and M‐MgAl‐700 were selected for the performance comparison with the extended synthesized magnetic LDHs and MMOs. It can be seen from Figure 3b that the order of removal capacities for Cd(II) and As(V) by all five types of mineralizers was as follows: M‐MgAl‐700 > M‐MgFeAl‐700 > M‐MgFe‐700 > M‐ZnAl‐700 ≈ M‐ZnFeAl‐700. The reasons for the above order of mineralization performance were closely related to the radius of metal ions, the pH value dispersed in water, as well as the content of defects. Thus, M‐MgAl‐700 was applied for a deeper investigation of its mineralization behaviors, performances, and mechanisms in the following research.

Detailed mineralizing tests of M‐MgAl‐700 towards single Cd(II) and As(V) were conducted. The results of the mineralization kinetics experiments (dosage = 1.0 g L^−1^, C_0_ = 500 and 50 mg L^−1^ for Cd(II) and As(V), pH ≈ 6.1 and 8.4, at room temperature) showed that, for Cd(II), it can be rapidly captured in 9 h with the removal efficiency > 99.0%, eventually reaching 99.7% at 48 h (Figure 3c). Besides, the data of prolonged mineralization tests shown in Figure S16 proved that the concentration of heavy metal ions can remain at a trace level and their removal efficiencies always exceeded 99.0% within one week, which supported the stability of the mineralization performance for M‐MgAl‐700. As shown in Figure 3d, the removal efficiency for individual As(V) can also reach over 99.0%, which indicated the fast capture rate for whether Cd(II) or As(V) by using M‐MgAl‐700. More importantly, the mineralizing behavior of M‐MgAl‐700 towards both Cd(II) and As(V) can be better fitted with the pseudo‐second‐order kinetics model (the correlation coefficient values (R^2^) > 0.99) than pseudo‐first‐order model, suggesting that this process was proceeded by chemisorption rather than physisorption (Figure 3e; Figure S17 and Table S2) [44]. In addition, the linear fitting of the intraparticle diffusion model for Cd(II) and As(V) mineralization by M‐MgAl‐700 was also shown in Figure S18 and Table S3, demonstrating that the removal process can be divided into two (surface diffusion and adsorption equilibrium) instead of three steps [45]. Possible reasons were inferred as follows: 1) The mineralization process was much rapid that the intraparticle diffusion was not the rate‐limiting step [46]. These heavy metal ions may penetrate instantaneously and then occupy the main active sites of M‐MgAl‐700, to the extent that the classical and slow “intraparticle diffusion” control stage cannot be seen at the temporal resolution of the actual experiments. 2) For LDHs (a type of mesoporous material), if the adsorption mainly occurred on the outer surface or within the large pores, then there was virtually no “intraparticle diffusion” resistance in the traditional sense.

Additionally, the stability test of M‐MgAl‐700 was carried out in deionized water (DI), single Cd(II) and As(V) solution, respectively. In Figure 3f, it can be found that none of Mg(II), Al(III), and Fe(III) in DI dissolved from M‐MgAl‐700 within 48 h, which was sufficient to prove the great stability of M‐MgAl‐700. Whether in Cd(II) or As(V) solution, only the Mg(II) ion released (112.36 and 10.91 mg g^‒1^, respectively), while both Al(III) and Fe(III) were retained in M‐MgAl‐700, dissolving trace amounts (< 0.5 mg g^‒1^), which manifested that the M‐MgAl‐700 mineralizer can act as a dual‐effect magnesium fertilizer to promote the growth of crops when applying it for the co‐contaminated soil in the subsequent experiments [47]: 1) simultaneous mineralization of multiple heavy metals; 2) targeted magnesium supply.

Furthermore, the maximum saturated mineralization capacities (Q_m_) of M‐MgAl‐700 towards individual Cd(II) and As(V) were explored by the mineralization isotherm investigation, respectively. Compared with the Freundlich isotherm model, the relevant fitting plots and parameters for both Cd(II) and As(V) can be more in line with the Langmuir model, with the R^2^ value of 0.9927 and 0.9976, respectively, elucidating the monolayer adsorption process upon M‐MgAl‐700 (Figure 3g,h; Figure S19 and Table S4) [48]. In accordance with the Langmuir model, the Q_m_ value of Cd(II) and As(V) can be calculated as 1173.80 and 93.11 mg g^‒1^, respectively, outperforming the majority of previous reported mineralizers so far, which was shown in Figure 3i and Table S5. Since the process of mineralizing heavy metal ions by using the metallic oxide (LDO, MgO, etc.) was often accompanied by its own dissolution‐reconstitution or phase chemical reactions [49], the Q_m_ would show a significant increase compared to other commonly used mineralizers (activated carbon, clay minerals, hydroxides, etc.).

### 2.4 Products Analysis and Mechanism Illustration

**FIGURE 4:**
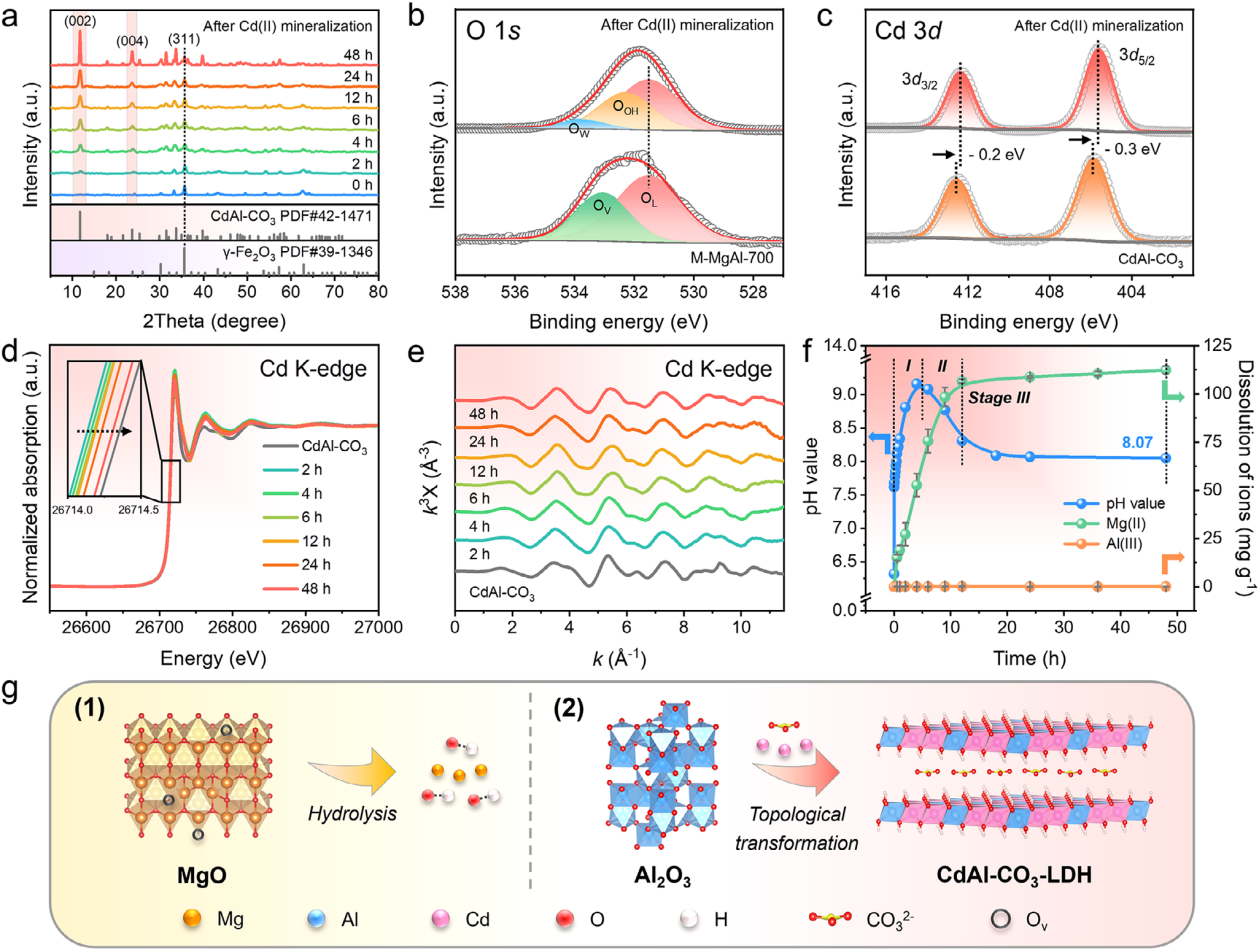
The characterization of M‐MgAl‐700 after Cd(II) mineralization, kinetics study, and proposed mechanism illustration. (a) The XRD patterns of M‐MgAl‐700 during Cd(II) removal process in different times; (b) O 1*s* and (c) Cd 3*d* XPS spectra of CdAl‐CO_3_, M‐MgAl‐700 before and after Cd(II) mineralization, respectively; (d, e) Cd K‐edge *k* ^3^χ (*k*) oscillation spectra for CdAl‐CO_3_ and M‐MgAl‐700 after Cd(II) mineralization (denoted as 2, 4, 6, 12, 24, and 48 h) and its corresponding *k*‐space data; (f) Monitoring of pH value in solution and dissolution kinetics behaviors of Mg(II) and Al(III) from M‐MgAl‐700; (g) Schematic diagram of the mineralization mechanism for Cd(II) using M‐MgAl‐700.

Regarding Cd(II), the mineralization process was monitored using a suite of quasi in situ characterization techniques (XRD, XPS, XAFS, etc.). As illustrated in Figure 4a, starting from 2 h, characteristic diffraction peaks belonging to the (00*l*) and other typical crystal planes of CdAl‐CO_3_ (PDF#42‐1471) began to appear [7]. With time progressed, the intensity of these peaks gradually increased, which revealed that the crystal structure of CdAl‐CO_3_ matured over time. Additionally, during this period, the intensity of the characteristic peaks of *γ*‐Fe_2_O_3_ stayed unchanged, demonstrating the high stability of the part of the magnetic microspheres. Apart from that, in FT‐IR spectra, the peaks at 3450, 1370, and < 1000 cm^‒1^ originated to the characteristic vibration of O‒H, CO_3_^2‒^, and M‒O [50], respectively, which further indicated the formation of CO_3_^2‒^ intercalated LDHs (Figure S20). Moreover, HRTEM images in Figure S21 displayed the lattice spacing of the nanosheets after Cd(II) mineralization was 2.67 Å, corresponding to the distance between (112) crystal facets of CdAl‐CO_3_ [51]. In Figure S22, on account of the positive charge imparted to the laminates of newly formed CdAl‐CO_3_, the zeta potential of the product increased from 18.97 to 23.13 before and after reaction, respectively.

Beyond that, XPS characterization pointed to the elemental species and electron transfer of products. After Cd(II) mineralization, the O 1*s* XPS spectrum can be fitted into three deconvoluted peaks attributed to the O_L_, O_OH_, and O_W_ at 531.5, 532.3, and 533.7 eV, respectively, which provided the supporting evidence that the oxygen defects in M‐MgAl‐700 were almost filled and subsequently converted into CdAl‐CO_3_ during the removal process in aqueous solution (Figure 4b). In the Cd 3*d* XPS spectra of Figure 4c, the binding energy of mineralization product showed a negative shift of ∼0.3 eV in comparison with the standard sample (CdAl‐CO_3_), which implied that the *γ*‐Fe_2_O_3_ and CdAl‐CO_3_ in the removal product were not merely physically mixed but interacted with each other, thereby exhibiting the strong electron transfer. Similar results were deeper corroborated by the XAFS spectroscopy. From the Cd K‐edge x‐ray absorption near‐edge structure (XANES), it can be first observed that the peak intensities of mineralized products obtained at different removal times manifested a slight difference, proving the distinction in the electronic structures of these samples (Figure 4d) [8]. Specifically, the absorption edge position of Cd exhibited an upward trend with the advancement of the reaction time and approached that of CdAl‐CO_3_, demonstrating that the oxidation state of Cd rose incrementally over time [52], and the crystal phase of products progressively tended to grow in a more complete manner, which kept the consistence with the results obtained from XPS. In Cd *k*‐space of Figure 4e, it was evident that the mineralized product possessed very close vibrational frequency with CdAl‐CO_3_ only at 2 h of the reaction, indicating that the Cd coordination structure of the product were similar to that of CdAl‐CO_3_ [53], and it began to emerge at an extremely early stage of the removal process, which was also consistent with the XRD results.

To thoroughly confirm the generation mechanism of CdAl‐CO_3_, M‐MgAl‐700 was dispersed in DI, whose stability was tested by quasi in situ XRD (Figure S23). Within 12 h, there were no characteristic peaks ascribed to LDHs that appeared in the XRD patterns of the M‐MgAl‐700, revealing that it cannot be restored to the corresponding M‐MgAl‐LDH. Briefly, the formation of CdAl‐CO_3_ was not achieved through the restoration of M‐MgAl‐700 and the occurrence of isomorphic substitution. Combined with the results shown in Figure 4a–e, it can be inferred that CdAl‐CO_3_ was directly formed in the solution at the beginning of the mineralization reaction. Further monitoring was conducted on the changes in the pH value of solution and the release kinetics behaviors of different metal cations during Cd(II) mineralization process, which can be divided into three stages: 1) 0–5 h: The MgO in M‐MgAl‐700 underwent hydrolysis reaction, which released a large amount of Mg(II) and OH^‒^, leading to the significant alkalization (from 6.3 to 9.2) of the surrounding aqueous environment (Equation 1). 2) 5–12 h: The pH value dropped to below 8.3 since the OH^‒^ was consumed in large quantities. Together with the Al_2_O_3_, Cd(II), CO_3_^2‒^, and H_2_O, they reacted to form CdAl‐CO_3_, causing the equilibrium of hydrolysis to shift in the forward direction, continuously releasing Mg(II) (Equation 2). 3) 12–48 h: The mineralizing reaction reached equilibrium with all parameters remaining stable. Interestingly, no release of Al(III) was detected throughout the entire process (< 0.15 mg L^‒1^), which validated that it was always present in the solid phase and played a crucial role in the in situ mineralization of Cd(II) (Figure 4f). Overall, the integrated schematic diagram in Figure 4g illustrated the removal mechanism for Cd(II) by which the topological transformation resulted in the in situ generation of CdAl‐CO_3_.

(1) $$\mathrm{MgO}+H_{2}O\to \mathrm{Mg}{\left(\mathrm{OH}\right)}_{2}\to {\mathrm{Mg}}^{2+}+2{\mathrm{OH}}^{-}$$

(2) $$\begin{array}{ccc} & & 3Al_{2}O_{3}+12Cd^{2+}+18OH^{-}+3{\mathrm{CO}}_{3}^{2-}+12H_{2}O\\ & & \;\to Cd_{12}Al_{6}{\left(\mathrm{OH}\right)}_{36}{\left(CO_{3}\right)}_{3}\cdot 3H_{2}O \end{array}$$

**FIGURE 5:**
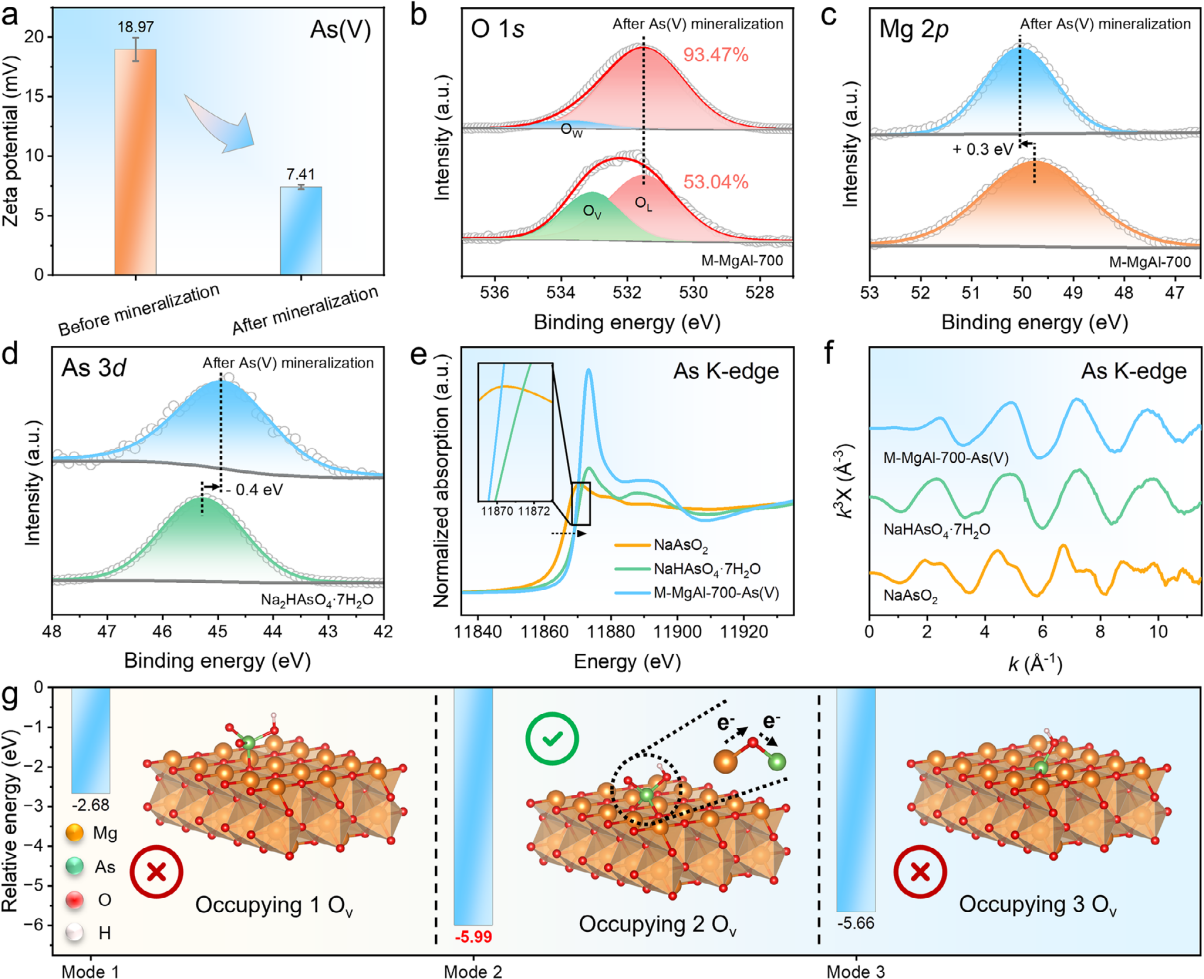
The characterization of M‐MgAl‐700 after As(V) mineralization, DFT calculation, and proposed mechanism illustration. (a) The zeta potential diagram of M‐MgAl‐700 before and after As(V) mineralization; (b) O 1*s*, (c) Mg 2*p*, and (d) As 3*d* XPS spectra of Na_2_HAsO_4_·7H_2_O, M‐MgAl‐700 before and after As(V) mineralization; (e, f) As K‐edge *k* ^3^χ (*k*) oscillation spectra for NaAsO_2_, Na_2_HAsO_4_·7H_2_O, and M‐MgAl‐700 after As(V) mineralization (denoted as M‐MgAl‐700‐As(V)) and its corresponding *k*‐space data; (g) Relative energies of As(V) mineralization by M‐MgAl‐700 in three modes (Inset: possible mineralization modes from the front view).

Concerning As(V), XRD, zeta potential, XPS, and other characterization jointly verified that the surface adsorption dominated the entire mineralization process. To be specific, there was no obvious change in the XRD pattern before and after reaction, which suggested that the structure of M‐MgAl‐700 was maintained and not affected by the As(V) oxygenated anion (Figure S24). As shown in Figure 5a, after As(V) removal, the zeta potential of the mineralized product showed a sharp decline trend from 18.97 to 7.41, indicating that As(V) was adsorbed on the surface and neutralized the original positive charge of M‐MgAl‐700 [10]. In the O 1*s* XPS spectra of Figure 5b, the peak of O_V_ disappeared after mineralization, supporting that the surface adsorption of As(V) was achieved by occupying the oxygen vacancies of M‐MgAl‐700 [11], which caused a marked increment of the O_L_ content (from 53.04% to 93.47%). Moreover, the Mg 2*p*, Al 2*p*, and As 3*d* XPS spectra were fitted and analyzed, respectively (Figure 5c,d; Figure S25). After As(V) anions were captured onto the surface of M‐MgAl‐700, the binding energies shifted by +0.3, +0.5, and −0.4 eV for Mg, Al, and As, in the same order, manifesting that electrons transferred from Mg and Al through O to As, which induced the valence state of As to be slightly reduced than that of the standard sample (Na_2_HAsO_4_·7H_2_O). Likewise, in the As K‐edge XANES, the absorption edge position of the product (denoted as M‐MgAl‐700‐As) was lower than that of Na_2_HAsO_4_·7H_2_O, which was in agreement with the results of XPS (Figure 5e). In Figure 5f, the As *k*‐space of demonstrated that M‐MgAl‐700‐As exhibited similar oscillations to Na_2_HAsO_4_·7H_2_O rather than Na_2_AsO_2_, revealing that the coordinated structure of As remained unaltered after the mineralization reaction.

To clarify the most stable adsorption modes of As(V) (HAsO_4_^2‒^) on M‐MgAl‐700, the DFT calculation was used to investigate the adsorption energy changes of HAsO_4_^2‒^ at three distinct modes (occupying 1, 2, or 3 O_V_, respectively) [54]. Firstly, the MgO (110) surface was modeled to simulate the prepared M‐MgAl‐700. The front and top views of possible models were shown in Figure 5g and Figure S27. The relative energies for the three modes of M‐MgAl‐700 were −2.68, −5.99, and −5.66 eV, respectively, proving that the binuclear bidentate adsorption mode possessed the highest stability since it had achieved the best balance among total bonding energy, charge balance, and geometric compatibility in comparison with mononuclear monodentate and trinuclear tridentate modes [55]. Collectively, the mineralization mechanism of surface adsorption towards HAsO_4_^2‒^ occurred via occupation of two oxygen vacancies per anion on the laminate of M‐MgAl‐700, which ensured its stable and effective removal.

### 2.5 Simultaneous Removal Performance in Water and Soil

**FIGURE 6:**
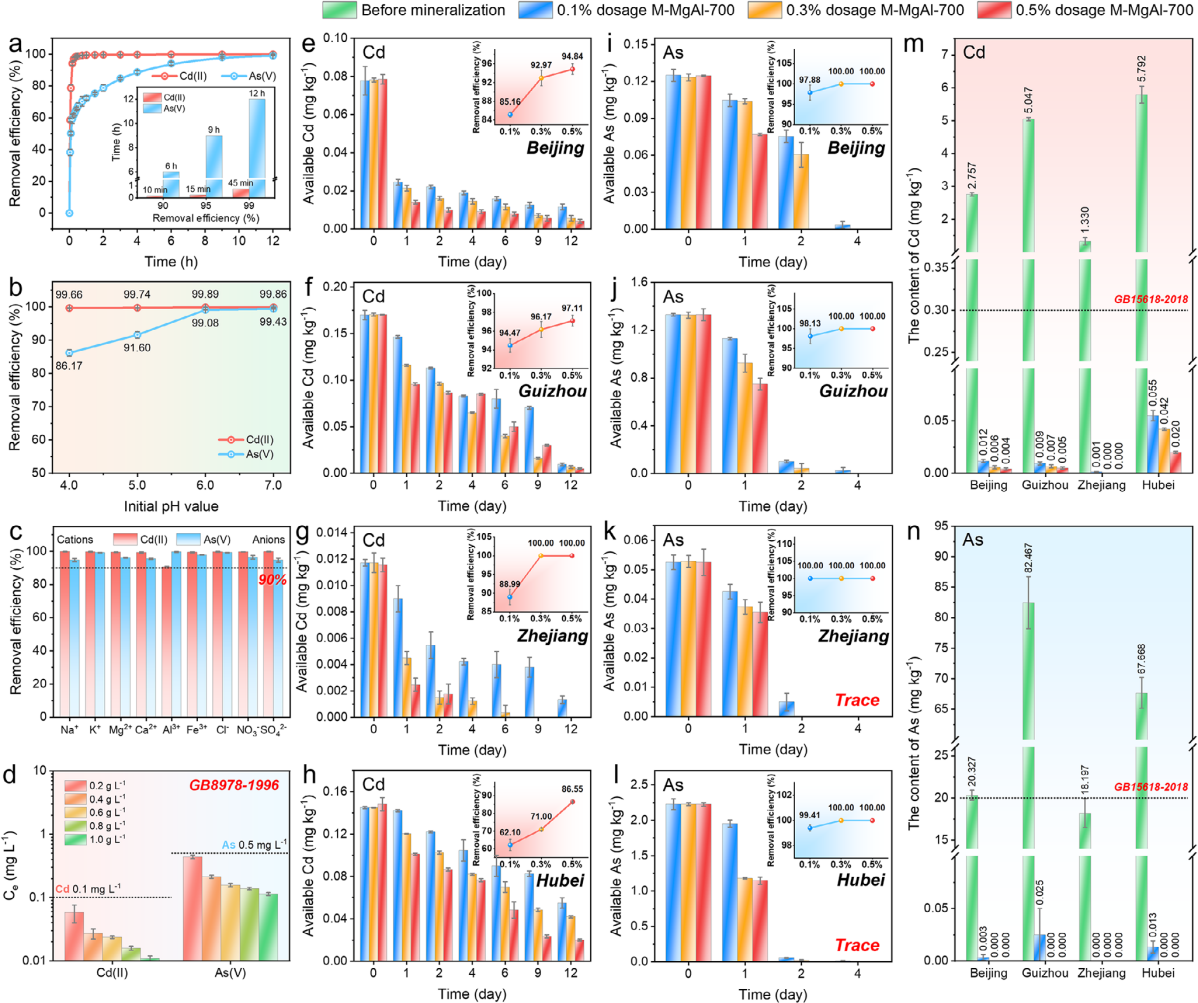
The removal performance of M‐MgAl‐700 towards coexisting Cd(II) and As(V) in water and soil. (a) The simultaneous mineralization experiments using M‐MgAl‐700 in coexisting Cd(II) and As(V) aqueous solution with initial concentration for each ion of 20 mg L^−1^ and pH value of 6.0 (Inset: time required to achieve different removal efficiencies); Comparison of the removal efficiencies for Cd(II) and As(V) under (b) different initial pH values and (c) different coexisting ions, respectively; (d) Comparison of C_e_ by M‐MgAl‐700 with different dosages; Content of the available (e–h) Cd and (i–l) As by M‐MgAl‐700 in the simulated co‐contaminated soil similar to that in Beijing, Guizhou, Zhejiang, and Hubei during different mineralization times, respectively (Inset: removal efficiencies of M‐MgAl‐700 with different dosages); Comparison of the residual available (m) Cd and (n) As.

In the first place, M‐MgAl‐700 exhibited highly efficient, rapid, and stable removal performance for both Cd(II) and As(V) in the coexisting solution. In a solution with an initial concentration for each ion of 20 mg L^‒1^ and the pH value of 6.0, the removal rate can exceed 90.0%, 95.0%, and 99.0% within 10, 15, and 45 min for Cd(II) and 6, 9, and 12 h for As(V), illustrating that both two ions can be synchronously removed by M‐MgAl‐700, and the removal efficiency towards Cd(II) was even faster than As(V) (Figure 6a). Moreover, such a high removal efficiency can be maintained for over a week during the stability test without causing any secondary contamination (Figure S28). As shown in Figure S29, XPS survey spectra confirmed the simultaneous presence of two elements, proving that they were captured in M‐MgAl‐700. In order to simulate the actual acidity of leachate from acidic soil in southern China, the mineralizing experiments with other initial pH values of 4.0, 5.0, and 7.0 were also conducted, which showed similar phenomena in Figure S30. The summary curves of removal efficiencies for both Cd(II) and As(V) were presented in Figure 6b. It was found that M‐MgAl‐700 still maintained the removal rate of over 99.5% towards Cd(II) even in a more acidic environment, while for As(V), it exhibited a slight decrease but can remain > 85.0%. The reason was speculated to be the competitive adsorption of the other anions on the laminate. Notably, the fitting results of the adsorption kinetics model suggested that in the binary system, the mineralizing process for Cd(II) and As(V) onto M‐MgAl‐700 continued following the pseudo‐second‐order model, being indicative of chemisorption (Figure S31 and Table S6) [17].

In addition, the impact of large amounts of anions or cations presented in real water and/or soil on the mineralization performance was also explored in Figure 6c and Figure S32. Whether there were various cations (Na^+^, K^+^, Mg^2+^, Ca^2+^, Al^3+^, and Fe^3+^), distinct anions (Cl^‒^, NO_3_^‒^, SO_4_^2‒^, and PO_4_^3‒^), organics (humic acid (HA)), or other common heavy metals (Pb(II), Cu(II), Ni(II), As(III), and Cr(VI)) coexisting with Cd(II) and As(V), the removal rates were consistently retained above 90.0%, which demonstrated the excellent selectivity of M‐MgAl‐700 for both two ions. Among them, the removal efficiency of Cd(II) slightly decreased only when the Al^3+^ was additionally added. The speculated reason was that the free Al^3+^ and the already released Mg^2+^ in the system led to the tendency of generating MgAl‐LDH or Al(OH)_3_, thereby inhibiting the fixation of Cd(II). On the other hand, compared with Cl^−^, the SO_4_^2−^ and NO_3_^−^ showed similar characteristics to HAsO_4_^2−^, containing a large number of oxygen atoms and being prone to competing with HAsO_4_^2−^ for the oxygen vacancies on the surface of M‐MgAl‐700, so the removal efficiency and selectivity of As(V) decreased marginally. Furthermore, the residual contents of Cd(II) and As(V) in aqueous solution were monitored in Figure 6d, which showed that it can be reduced to levels compliant with GB8978‐1996 for sewage discharge with the dosage of only 0.2 g L^‒1^, combining both efficiency and cost.

For the investigation of the remediation performance for the co‐contaminated soil, the soil from Yanqing District (Beijing City), Guiyang City (Guizhou Province), Lanxi City (Zhejiang Province), and Huangshi City (Hubei Province) was simulated to verify the universality of M‐MgAl‐700. The contents of heavy metal ions and other naturally occurring anions (PO_4_^3−^, Cl^‒^, and SO_4_^2−^, etc.) in simulated soil was preprocessed and tested by ICP‐OES and Ion Chromatography (IC), in which the actual measured data of Cd and As did not differ greatly from theoretical values, and the contents of the other ions were all within the normal range, so that the soil can be applied for the subsequent experiments (Tables S7 and S8). M‐MgAl‐700 was added to the soil with the three dosages of 0.1%, 0.3%, and 0.5%, and the available Cd and As were detected before and after mineralization. As displayed in Figure 6e–h, in multiple sets of parallel experiments, the available Cd decreased over time and eventually reached the maximum after 12 days, with the removal efficiencies achieving above 85.0% when the dosage of M‐MgAl‐700 was 0.5%. On the other hand, the removal of available As was much more rapid, which can be depleted to a trace level in only 4 days, with the removal rate approaching 100.0% (Figure 6i–l). Taken together, it can be seen that in terms of the available Cd, the dosage of M‐MgAl‐700 possessed a greater impact on its removal efficiency, while for As, the influence was slighter. In future actual applications, “menu‐style customization” for the dosage of mineralizer can be determined based on the actual concentrations of available heavy metals in soil [5]. As compared to the GB15618‐2018 standard in Figure 6m,n, whether for Cd(II) or As(V), their contents in the soil after remediation were far below, which can be utilized for agricultural land.

### 2.6 Magnetic Separation Test

**FIGURE 7:**
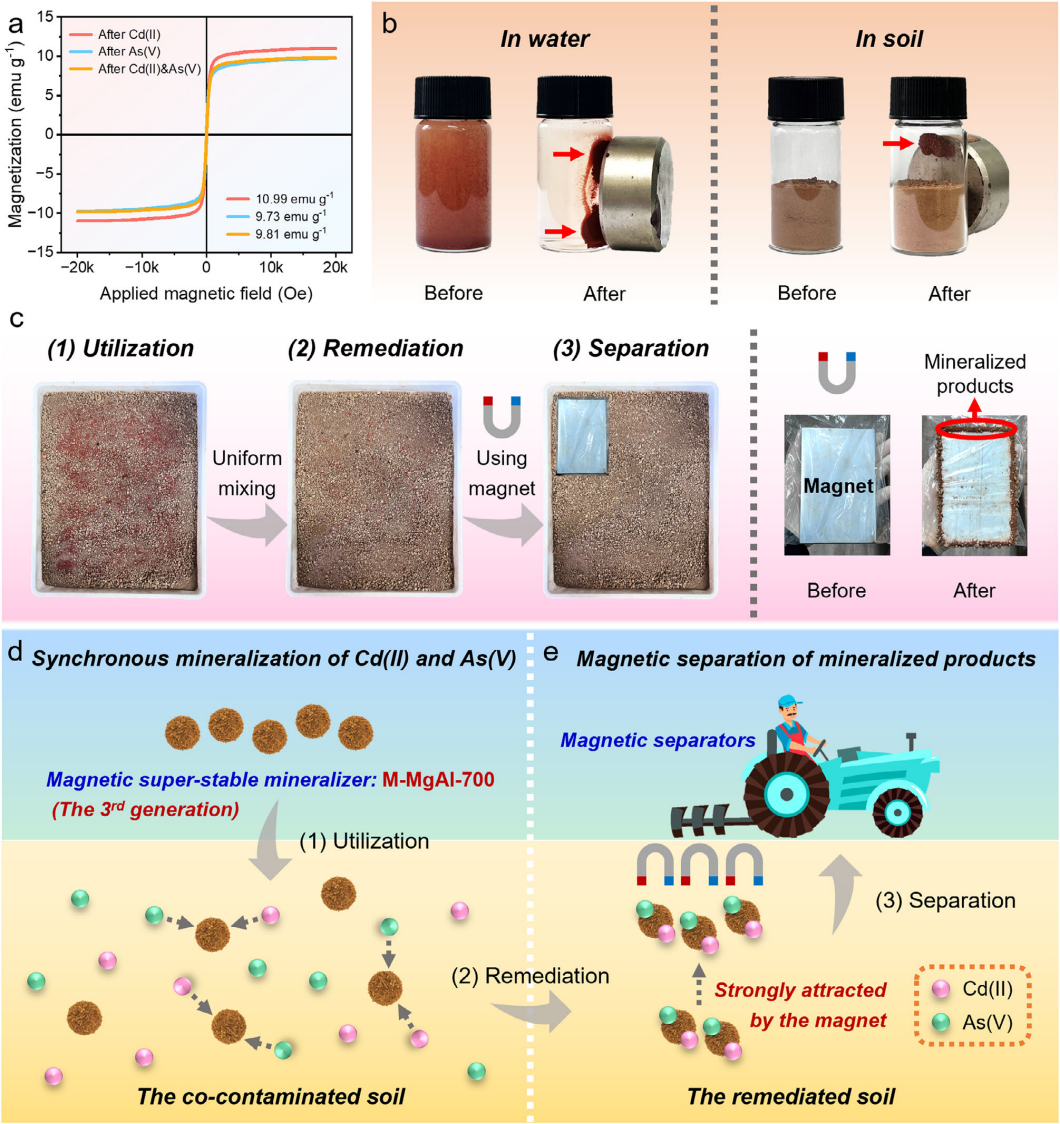
The characterization of magnetic properties for mineralized products. (a) The room‐temperature magnetization hysteresis loops of mineralized products; (b) Images of the magnetic separation in water and soil at the laboratory scale; (c) Simulated experimental images and (d,e) schematic diagram of utilizing the third‐generation magnetic super‐stable mineralizer (M‐MgAl‐700) for co‐contaminated soil remediation and magnetic separation in practical application.

After mineralizing Cd(II) and/or As(V), the spent M‐MgAl‐700 still maintained adequate magnetism. In Figure 7a, the saturation magnetization values of mineralized products remained at ∼10.0 emu g^‒1^, and the shape of the curves indicated that they were still superparamagnetic [56]. When M‐MgAl‐700 microspheres were applied for the treatment of actual wastewater, they can be evenly distributed by vigorous shaking or sonication to generate a brownish‐red suspension (Figure 7b). Rapid aggregation (< 10 s) of the products from their homogeneous dispersion was found upon application of an external magnetic field (employing a NdFeB magnet). Conversely, the products can be quickly redispersed with gentle shaking once the magnet is removed. Similar effects can also be achieved in the soil environment.

Building on this, the simulation experiments were conducted at a scale suitable for practical applications, as shown in Figure 7c. The large‐sized pallet was utilized as the farmland, with a flat‐shaped strong magnet to simulate the magnetic vehicle that can operate in the field. To begin with, the third‐generation magnetic super‐stable mineralizer (take M‐MgAl‐700 for example) was uniformly dispersed in the co‐contaminated soil. Subsequently, give a period of time to achieve in situ mineralization of multiple heavy metals (e.g., Cd(II), As(V), etc.). Afterwards, the magnet was used to move in a specific direction to completely separate the recycled mineralizer from the soil. It can be easily observed that a large amount of solid powder was attached to the magnet, namely M‐MgAl‐700. By testing the quality of the M‐MgAl‐700 before and after mineralization, respectively, it can be calculated that the separation efficiency exceeded 90.0% (Figure S33). The schematic diagram of the corresponding steps was presented in Figure 7d,e. The above result corroborated that the third‐generation magnetic super‐stable mineralizer possessed the dual advantages of both high performance and convenience.

## 3 Conclusion

Following the first‐generation and second‐generation heavy metal mineralizers, the third‐generation magnetic super‐stable mineralizer was innovatively developed by carrying out high‐temperature calcination of the magnetic LDHs precursors, which possessed both satisfactory removal performance and complete separation for coexisting heavy metal cations and anions in wastewater and contaminated soil. Through the regulation of synthesis conditions, including the metal element and temperature, it can be extended to prepare a range of magnetic structured mineralizers, highlighting the generality of the approach. The representative M‐MgAl‐700 showed a typical core‐shell structure with a size of *c.a*. 500 nm, featuring high superparamagnetism, a large specific surface area, and abundant oxygen vacancies. M‐MgAl‐700 exhibited high maximum saturated mineralization capacities of 1173.80 and 93.11 mg g^‒1^ towards single Cd(II) and As(V) in solution, respectively, surpassing most reported mineralizers. Both the corresponding mineralization data can be well fitted with pseudo‐second‐order kinetics, intraparticle diffusion, and Langmuir isotherm models, which manifested the process of monolayer chemisorption. In the sewage polluted by coexisting Cd(II) and As(V), both contents can be reduced to the levels below the limit specified in the GB8978‐1996 standard by applying only 0.2 g L^‒1^ M‐MgAl‐700. As for the co‐contaminated soil in four distinct regions, 0.5% dosage of M‐MgAl‐700 can simultaneously immobilize > 85.0% and nearly 100.0% of available Cd and As after treatment for 4 and 12 days, respectively, whose contents can fall far below the GB15618‐2018 standard for agricultural land. The removal mechanisms were elucidated by quasi in situ XRD, XPS, XAFS, and DFT calculation, identified as the topological transformation into CdAl‐CO_3_ for Cd(II) and surface adsorption via the binuclear bidentate mode for the oxygenated anion of As(V), respectively. Moreover, the mineralized product persisted competent magnetism, which can be rapidly gathered within 10 s under an external magnetic field, resulting in the effect of thorough isolation. This research opened a new pathway for efficient removal and complete separation of multiple heavy metal pollutants in practical applications.

## 4 Experimental Section

### 4.1 Materials

All the chemicals (including FeCl_3_·6H_2_O, CH_3_COONa (NaAC), Mg(NO_3_)_2_·6H_2_O, Al(NO_3_)_3_·9H_2_O, NaOH, Na_2_CO_3_, Na_2_SO_4_, Na_2_HPO_4_·12H_2_O, KCl, CaCl_2_, NH_3_·6H_2_O, C_8_H_20_O_4_Si (ethyl silicate, abbreviated as TEOS), Cd(NO_3_)_2_·4H_2_O, Na_2_HAsO_4_∙7H_2_O, Pb(NO_3_)_2_, Cu(NO_3_)_2_·3H_2_O, Ni(NO_3_)_2_·6H_2_O, NaAsO_2_, K_2_Cr_2_O_7_ (require drying before use), C_6_H_15_NO_3_ (TEA), C_14_H_23_N_3_O_10_ (DTPA), nitric acid (HNO_3_), and humic acid (HA, C_9_H_9_NO_6_)) and solvents (methanol, ethanol, and glycol (EG)) were analytical reagent grade and required no further purification. DI was suitable to all experiments.

### 4.2 Synthesis

*γ*‐Fe_2_O_3_ was synthesized via a solvothermal method using EG as the solvent, by reacting FeCl_3_·6H_2_O and NaAC at 200°C for 12 h [24]. The product was magnetically separated, washed twice each with DI and ethanol, and dried at 60°C for further use.

*γ*‐Fe_2_O_3_@SiO_2_ was synthesized by a sol–gel method, which involved sequentially adding *γ*‐Fe_2_O_3_, NH_3_·6H_2_O, and TEOS into a mixed solution of DI and ethanol [22]. The mixture was ultrasonically stirred for 6 h. After that, the product was magnetically separated, washed twice each with DI and ethanol, and dried at 60°C for further use.

Under continuous ultrasonication, a salt solution (dissolving Mg(NO_3_)_2_·6H_2_O and Al(NO_3_)_3_·9H_2_O in DI) and an alkaline solution (dissolving NaOH and Na_2_CO_3_ in DI) were simultaneously added dropwise, enabling the in situ growth of MgAl‐LDH on the surface of *γ*‐Fe_2_O_3_@SiO_2_ while maintaining a constant pH of ∼10.0 [25]. The product was magnetically separated, washed twice each with DI and ethanol, and dried at 60°C for further use (denoted as M‐MgAl‐LDH).

The M‐MgAl‐LDH was calcined at temperatures ranging from 400°C to 900°C to obtain M‐MgAl‐X [26]. This process was carried out under a heating rate of 2°C min^‒1^, with a holding time of 3 h at each target temperature.

Similarly, by employing other divalent and trivalent metal salts (Zn(NO_3_)_2_·6H_2_O and Fe(NO_3_)_2_·6H_2_O) while keeping other conditions constant, the extended synthesis for all the M‐MgFeAl‐LDHs/MMOs, M‐MgFe‐LDHs/MMOs, M‐ZnAl‐LDHs/MMOs, and M‐ZnFeAl‐LDHs/MMOs can be achieved.

### 4.3 Mineralization Performance Study

By implementing the mechanical stirring to disperse 0.2–1.0 g L^−1^ of magnetic mineralizers in the solution of single or coexisting Cd(II) and/or As(V) with different concentrations (10–2000 mg L^−1^), the mineralization experiments were conducted for different durations at room temperature. At the regular intervals (0–48 h), 0.5–2 mL of the suspension was extracted and instantly filtrated through a 0.22 µm membrane. The contents of Cd, As, Mg, Al, and Fe in solution were analyzed by ICP‐OES.

The Cd and As co‐contaminated soil from four different regions was simulated by using the heavy metals mixed aqueous solution to immerse the blank soil for 12 h. The contaminated sample (50.0 g) was thoroughly mixed with 50, 150, and 250 mg M‐MgAl‐700 at room temperature, respectively (i.e. 0.1%, 0.3%, and 0.5% dosage). In all cases, the ratio of soil mixture to DI solution was 50 g to 40 mL. About 3.0 g soil sample was collected every two or three days and dried at 60°C for further use. The available Cd and As in samples were extracted by CaCl_2_ and buffered DTPA extraction methods, respectively, and the concentrations were quantified by ICP‐OES.

### 4.4 Characterization

SEM images and the corresponding EDX analytical data were obtained on a Zeiss Supra 55 SEM. HRTEM images were captured using a JEOL JEM‐2010 transmission electron microscope operating at an acceleration voltage of 200 kV. TG‐DTA were conducted on a STA‐449C Jupiter (HCT‐2 Corporation, China) and the samples were heated from 25 to 1000°C at a heating rate of 10°C min^−1^ under a N_2_ atmosphere (20 mL min^−1^). XRD patterns were characterized by a Rigaku XRD‐6000 diffractometer equipped with Cu Kα radiation (λ = 1.5405 Å). FT‐IR spectra were measured on a Nicolet 6700 (Thermo) in the range of 400–4000 cm^−1^ with samples prepared as KBr pellets. The zeta potential test was determined by Zetasizer Nano ZS. The magnetic properties of the samples were evaluated by utilizing a vibrating sample magnetometer (VSM, 7404, LakeShore, USA) at room temperature. N_2_ adsorption data were recorded at 77 K on a Quantachrome Autosorb‐1C analyzer, and the specific surface areas were derived from the adsorption data subjected to the BET method. XPS spectra were acquired by Monochromatic Al Kα exciting X‐radiation (PHI Quantera SXM) and calibrated against C 1*s* at 284.8 eV. XAFS data of Cd K‐edge and As K‐edge were collected at the 1W1B beamline of the Beijing Synchrotron Radiation Facility and the ID46(HEPS)‐X‐Ray Absorption Spectroscopy Beamline of High Energy Photon Source, respectively. The measurement was carried out in a transmission mode at room temperature, and its shell fitting was processed employing the Artemis Software.

### 4.5 Computational Details

The first principles density functional theory plus Hubbard U (DFT+U) calculation was performed by using the CASTEP module in the Material Studio software package. The Perdew‐Burke‐Ernzerhof (PBE) functional in generalized gradient approximation (GGA) was utilized to describe the exchange and correlation and potential in the single‐electron Kohn‐Sham equation. The ultrasoft pseudopotential was applied to describe the ionic cores to improve the transferability and reduce the number of plane waves required in the expansion of the Kohn‐Sham orbitals [57]. The potential energy surface was searched using the Broyden‐Fletcher‐Goldfarb‐Shanno (BFGS) algorithm. The cutoff energy was set as 381.0 eV to achieve a balance between cost and effectiveness. The geometry optimization was based on the following points: 1) an energy tolerance of 1.0 · 10^−4^ eV atom^−1^, 2) a maximum displacement tolerance of 2.0 × 10^−3^ Å, and 3) a maximum force tolerance of 0.05 eV Å^−1^. The k‐point meshes for the Brillouin zone integrations were 2 · 1 · 1 in the a, b, and c directions.

## Conflicts of Interest

The authors declare no conflicts of interest.

## Supporting information




**Supporting File**: advs75289‐sup‐0001‐SuppMat.docx.

## Data Availability

The data that support the findings of this study are available on request from the corresponding author. The data are not publicly available due to privacy or ethical restrictions.
