# Supplementary material for: The Third‐Generation Magnetic Super‐Stable Mineralizer: Complete Removal and Separation of Multiple Heavy Metal Pollutants
Source: Adv Sci (Weinh). 2026 Apr 15;13(39):e75289. doi: 10.1002/advs.75289 (PMC13335053; doi:10.1002/advs.75289)
Supplement: Supplementary file 1 — Supporting File: advs75289‐sup‐0001‐SuppMat.docx. [file ADVS-13-e75289-s001.docx]

***Supporting Information for***

**The third-generation magnetic super-stable mineralizer: Complete removal and separation of multiple heavy metal pollutants**

Haoran Wang,*^a^* Menghan Huang,*^a^* Ruihua Mao,*^a^* Xiaohan Zhang,*^a^* Zhaohui Wu,*^a^* Xiaofeng Pang,*^a^* Tong Lin,*^a^* Dongyuan Cui,*^a^* Sai An**^a^* and Yu-Fei Song**^a^*

**List of contents:**

**Figure S1.** SEM images of (a) γ-Fe_2_O_3_ and (b) γ-Fe_2_O_3_@SiO_2_.

**Figure S2.** HRTEM image of M-MgAl-LDH.

**Figure S3.** EDS line scanning data of M-MgAl-LDH.

**Figure S4.** EDS mapping data of M-MgAl-LDH.

**Figure S5.** TG curves of γ-Fe_2_O_3_, γ-Fe_2_O_3_@SiO_2_, MgAl-LDH, and M-MgAl-LDH.

**Figure S6.** XRD patterns of *γ*-Fe_2_O_3_, *γ*-Fe_2_O_3_@SiO_2_, M-MgAl-LDH, and M-MgAl-X.

**Figure S7.** Photographs of *γ*-Fe_2_O_3_, *γ*-Fe_2_O_3_@SiO_2_, M-MgAl-LDH, and M-MgAl-X.

**Figure S8.** FT-IR spectra of *γ*-Fe_2_O_3_, *γ*-Fe_2_O_3_@SiO_2_, M-MgAl-LDH, and M-MgAl-X.

**Figure S9.** Zeta potential diagram of *γ*-Fe_2_O_3_, *γ*-Fe_2_O_3_@SiO_2_, M-MgAl-LDH, and M-MgAl-X.

**Figure S10.** (a-e) Room-temperature magnetization hysteresis loops of M-MgAl-X (X = 400, 500, 600, 800, and 900); (f) Saturation magnetization values diagram of *γ*-Fe_2_O_3_, *γ*-Fe_2_O_3_@SiO_2_, M-MgAl-LDH, and M-MgAl-X.

**Figure S11.** N_2_ adsorption-desorption isotherms of M-MgAl-X (X = 400, 500, 600, 800, and 900).

**Figure S12.** O 1*s* XPS spectra of M-MgAl-LDH and M-MgAl-X.

**Figure R13.** (a) Fe 2*p*, (b) Mg 2*p*, and (c) Al 2*p* XPS spectra of γ-Fe_2_O_3_, MgAl-LDH, and M-MgAl-LDH.

**Figure S14.** XRD patterns of (a) MgFeAl-LDH and MgFe-LDH and (b) ZnAl-LDH and ZnFeAl-LDH.

**Figure S15.** Room-temperature magnetization hysteresis loops of the various magnetic LDHs and MMOs.

**Figure S16.** C_t_ and removal efficiencies of M-MgAl-700 in (a) Cd(II) and (b) As(V) solution with the different mineralization times.

**Figure S17.** Pseudo-first-order adsorption kinetic model fitted by linear regression of M-MgAl-700 for individual (a) Cd(II) and (b) As(V).

**Figure S18.** Intraparticle diffusion model fitted by linear regression of M-MgAl-700 for individual (a) Cd(II) and (b) As(V).

**Figure S19.** Freundlich adsorption isotherms model fitted by linear regression of M-MgAl-700 for individual (a) Cd(II) and (b) As(V).

**Figure S20.** FT-IR spectrum of M-MgAl-700 after Cd(II) mineralization.

**Figure S21.** HRTEM image of M-MgAl-700 after Cd(II) mineralization.

**Figure S22.** Zeta potential diagram of M-MgAl-700 before and after Cd(II) mineralization.

**Figure S23.** XRD patterns of M-MgAl-700 dispersed in DI in different times.

**Figure S24.** XRD patterns of M-MgAl-700 before and after As(V) mineralization.

**Figure S25.** Al 2*p* XPS spectra of M-MgAl-700 before and after As(V) mineralization.

**Figure S26.** As K-edge *k*^3^χ (*k*) oscillation spectra for NaAsO_2_, Na_2_HAsO_4_·7H_2_O, and M-MgAl-700 after As(V) mineralization (denoted as MgAl-700-As(V)) and its corresponding *R*-space data.

**Figure S27.** Possible modes of M-MgAl-700 after As(V) mineralization from the top view.

**Figure S28.** Removal efficiencies and C_t_ of M-MgAl-700 in coexisting Cd(II) and As(V) solution with the different mineralization times.

**Figure S29.** XPS survey spectra of M-MgAl-700 before and after Cd(II) and As(V) mineralization.

**Figure S30.** The simultaneous mineralization experiments using M-MgAl-700 in coexisting Cd(II) and As(V) aqueous solution with initial concentration for each ion of 20 mg L^−1^ and pH value of (a) 4.0, (b) 5.0, and (c) 7.0.

**Figure S31.** (a) Pseudo-first-order and (b, c) pseudo-second-order adsorption kinetic models fitted by linear regression of M-MgAl-700 for coexisting Cd(II) and As(V) with initial pH value of 6.0, respectively.

**Figure S32.** Comparison of the removal efficiencies for Cd(II) and As(V) under different coexisting ions.

**Figure S33.** The quality of the mineralizer/mineralized product during repeated five-time cycles in (a) water and (b) soil.

**Table S1.** The ion concentration of the solution and atomic ratio after digestion of M-MgAl-700.

**Table S2.** The fitting data of pseudo-first-order and pseudo-second-order adsorption kinetic models of M-MgAl-700 for individual Cd(II) and As(V).

**Table S3.** The fitting data of Interparticle diffusion adsorption kinetic models of M-MgAl-700 for individual Cd(II) and As(V).

**Table S4.** The fitting data of Langmuir and Freundlich adsorption isotherm models of M-MgAl-700 for individual Cd(II) and As(V).

**Table S5.** Comparison of removal performance for Cd(II) and As(V) by different mineralizers.

**Table S6.** The fitting data of pseudo-first-order and pseudo-second-order adsorption kinetic models of M-MgAl-700 for coexisting Cd(II) and As(V).

**Table S7.** Theoretical and actual values of the Cd(II) and As(V) concentrations in simulated polluted soils.

**Table S8.** Measured values of the contents of common anions in simulated polluted soils.

**Supporting Figures and Tables**


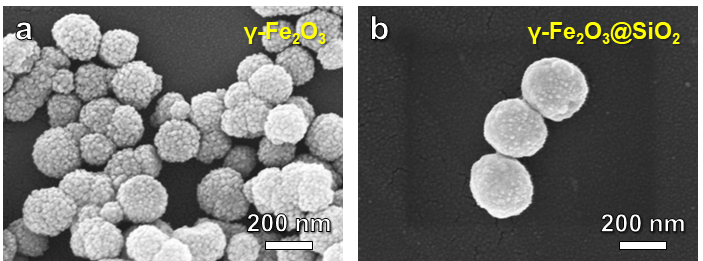


**Figure S1.** SEM images of (a) γ-Fe_2_O_3_ and (b) γ-Fe_2_O_3_@SiO_2_.


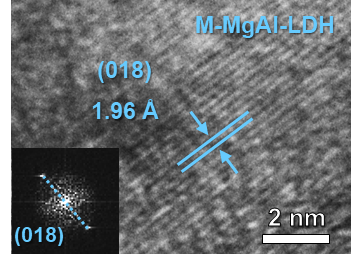


**Figure S2.** HRTEM image of M-MgAl-LDH.


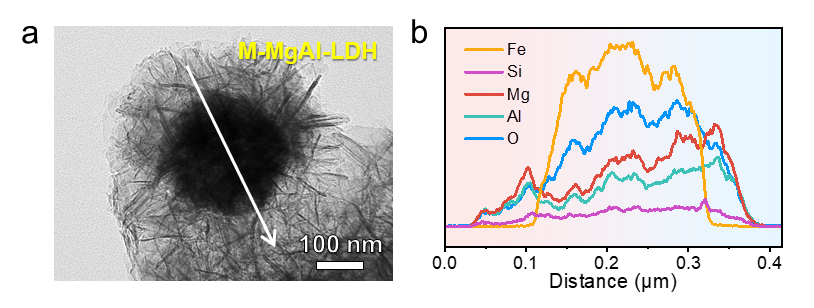


**Figure S3.** EDS line scanning data of M-MgAl-LDH.


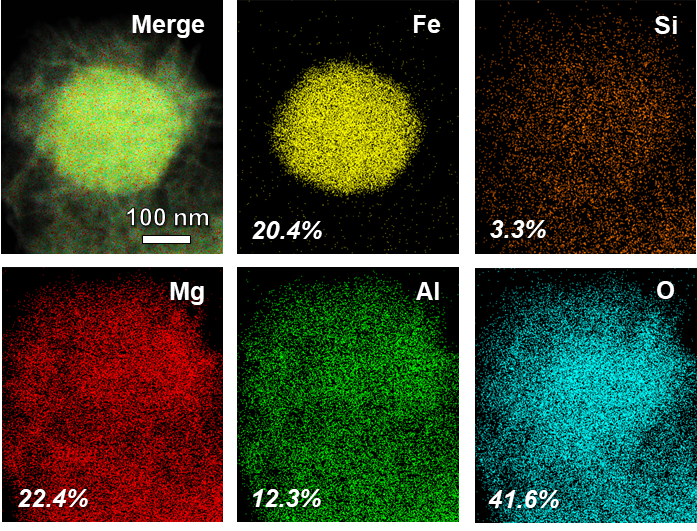


**Figure S4.** EDS mapping data of M-MgAl-LDH.





**Figure S5.** TG curves of γ-Fe_2_O_3_, γ-Fe_2_O_3_@SiO_2_, MgAl-LDH, and M-MgAl-LDH.


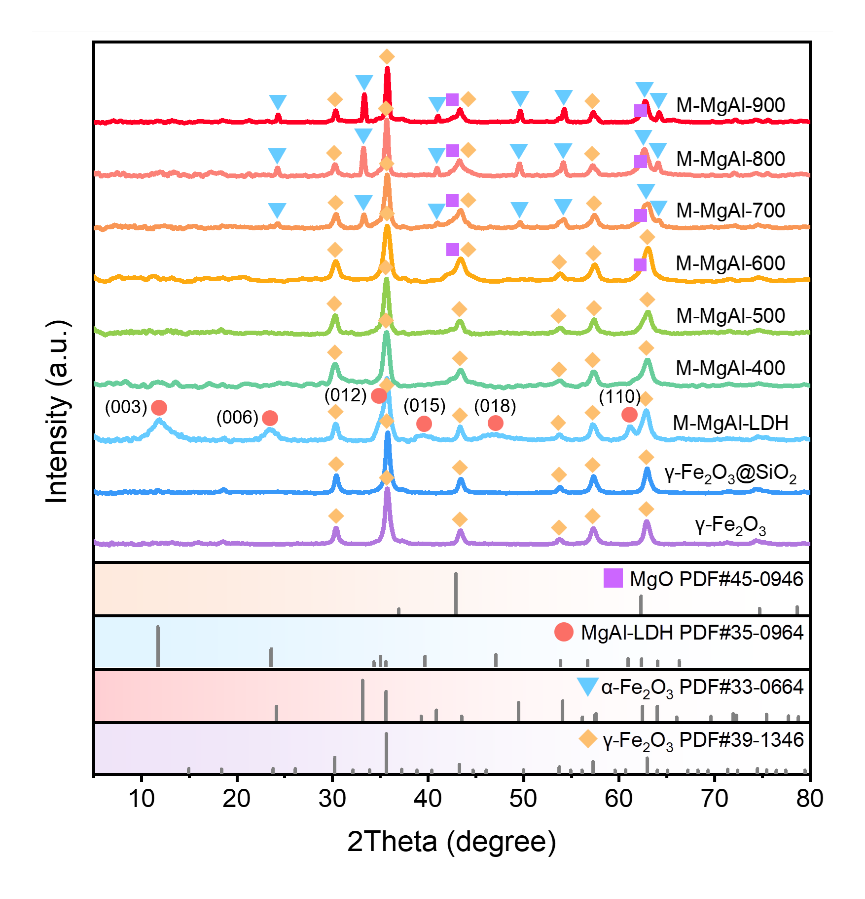


**Figure S6.** XRD patterns of *γ*-Fe_2_O_3_, *γ*-Fe_2_O_3_@SiO_2_, M-MgAl-LDH, and M-MgAl-X.


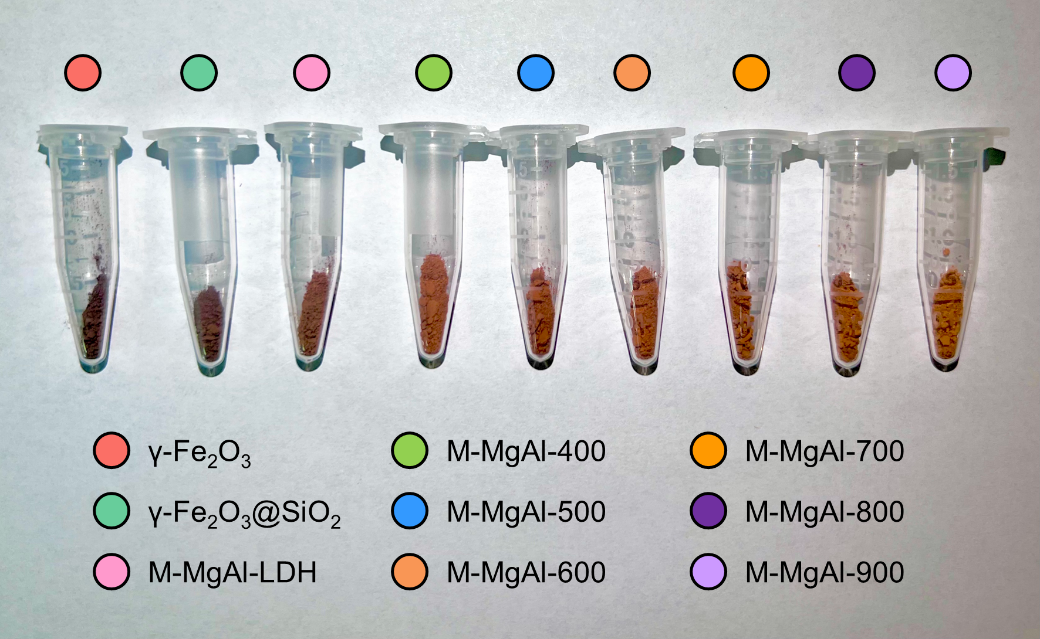


**Figure S7.** Photographs of *γ*-Fe_2_O_3_, *γ*-Fe_2_O_3_@SiO_2_, M-MgAl-LDH, and M-MgAl-X.





**Figure S8.** FT-IR spectra of *γ*-Fe_2_O_3_, *γ*-Fe_2_O_3_@SiO_2_, M-MgAl-LDH, and M-MgAl-X.





**Figure S9.** Zeta potential diagram of *γ*-Fe_2_O_3_, *γ*-Fe_2_O_3_@SiO_2_, M-MgAl-LDH, and M-MgAl-X.


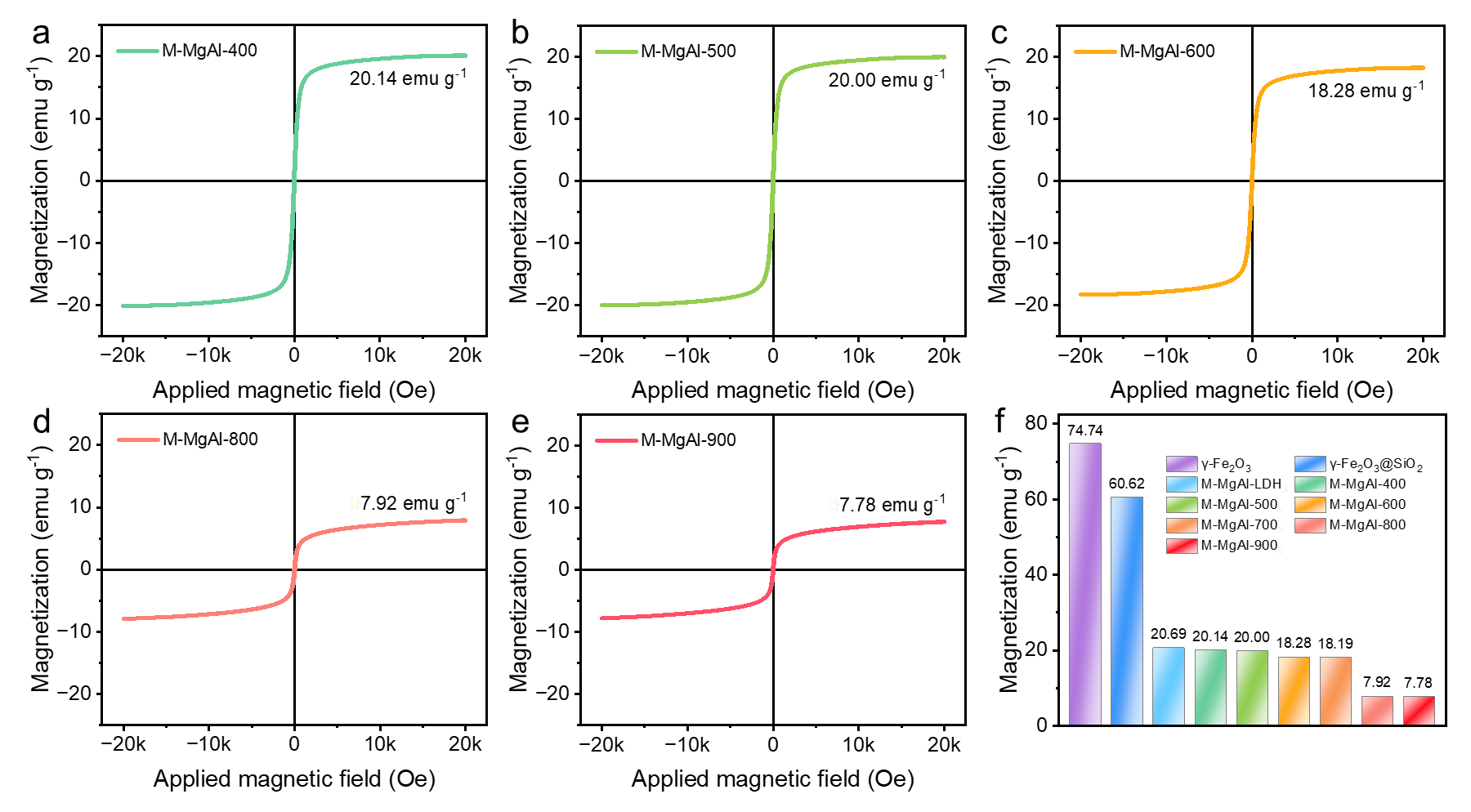


**Figure S10.** (a-e) Room-temperature magnetization hysteresis loops of M-MgAl-X (X = 400, 500, 600, 800, and 900); (f) Saturation magnetization values diagram of *γ*-Fe_2_O_3_, *γ*-Fe_2_O_3_@SiO_2_, M-MgAl-LDH, and M-MgAl-X.


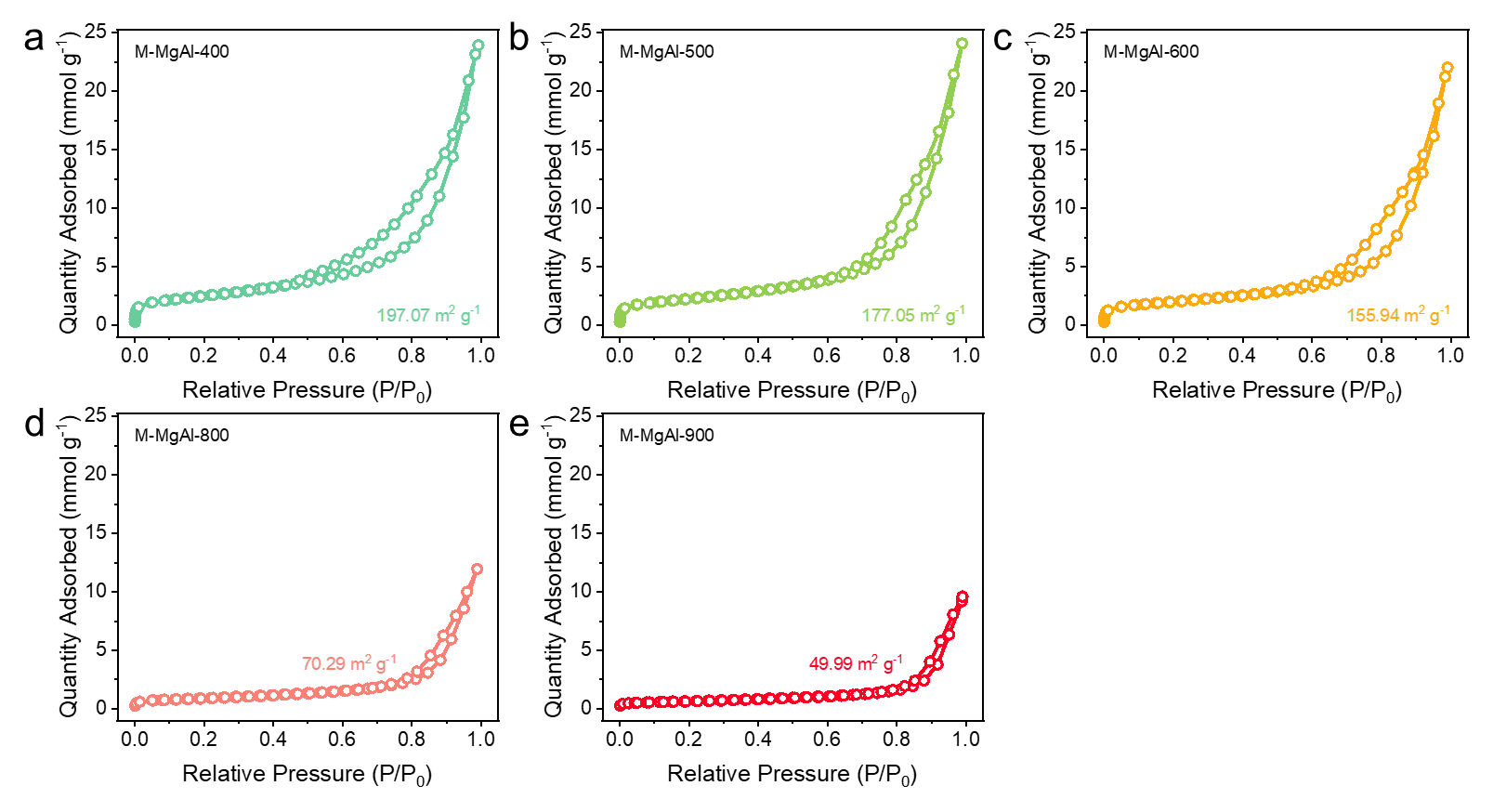


**Figure S11.** N_2_ adsorption-desorption isotherms of M-MgAl-X (X = 400, 500, 600, 800, and 900).


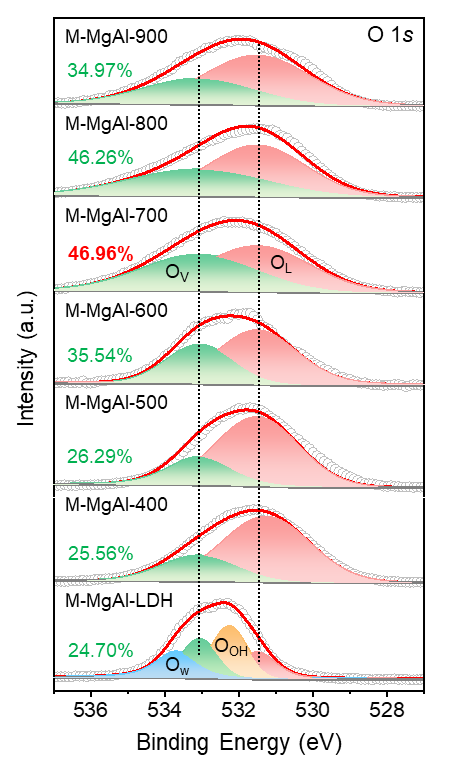


**Figure S12.** O 1*s* XPS spectra of M-MgAl-LDH and M-MgAl-X.

**
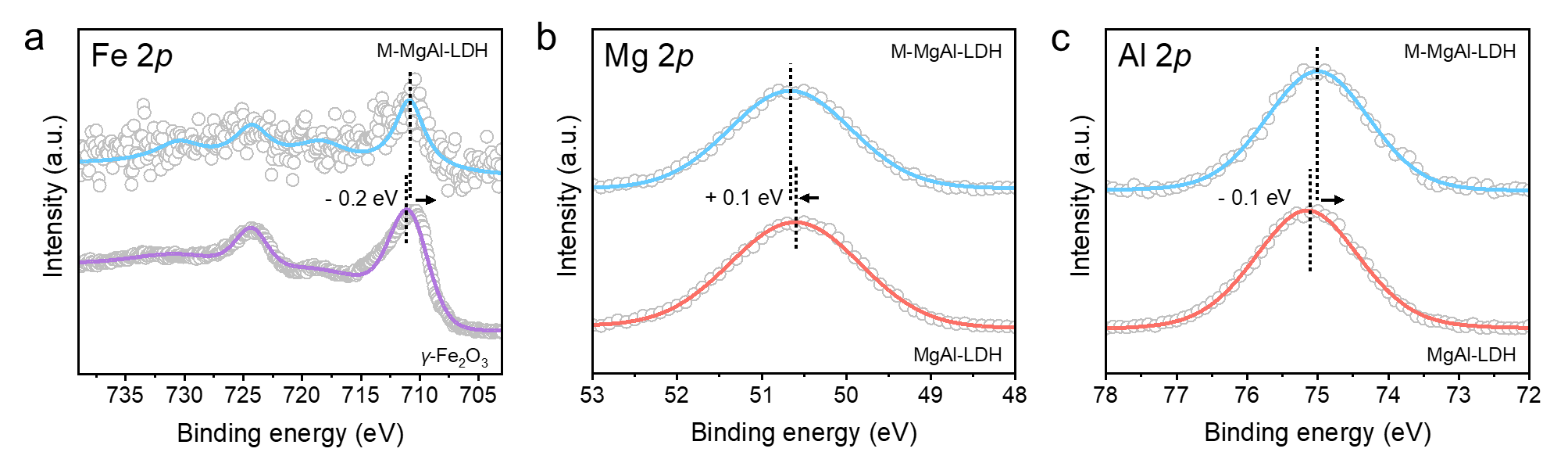
**

**Figure R13.** (a) Fe 2*p*, (b) Mg 2*p*, and (c) Al 2*p* XPS spectra of γ-Fe_2_O_3_, MgAl-LDH, and M-MgAl-LDH.


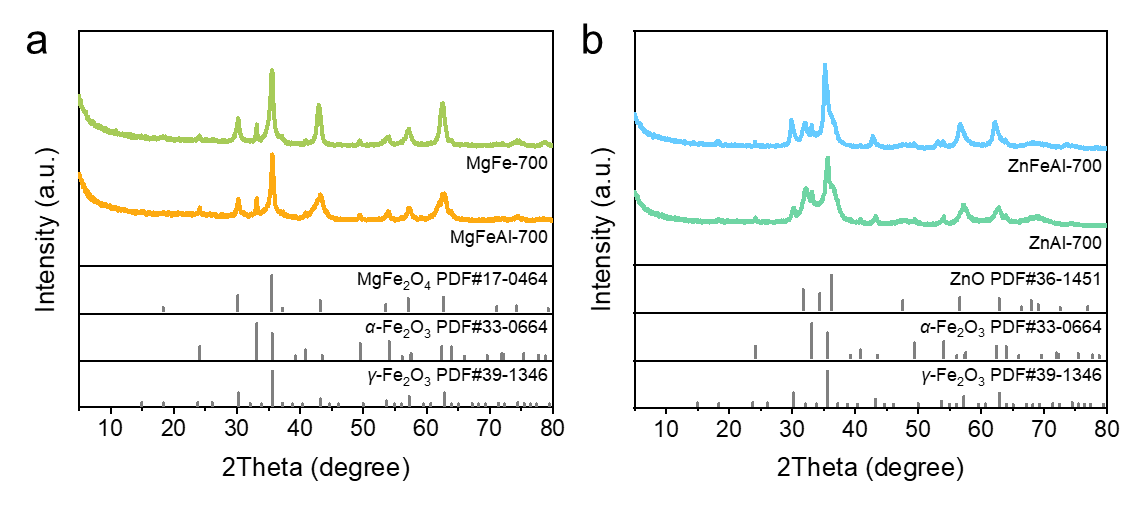


**Figure S14.** XRD patterns of (a) MgFeAl-LDH and MgFe-LDH and (b) ZnAl-LDH and ZnFeAl-LDH.


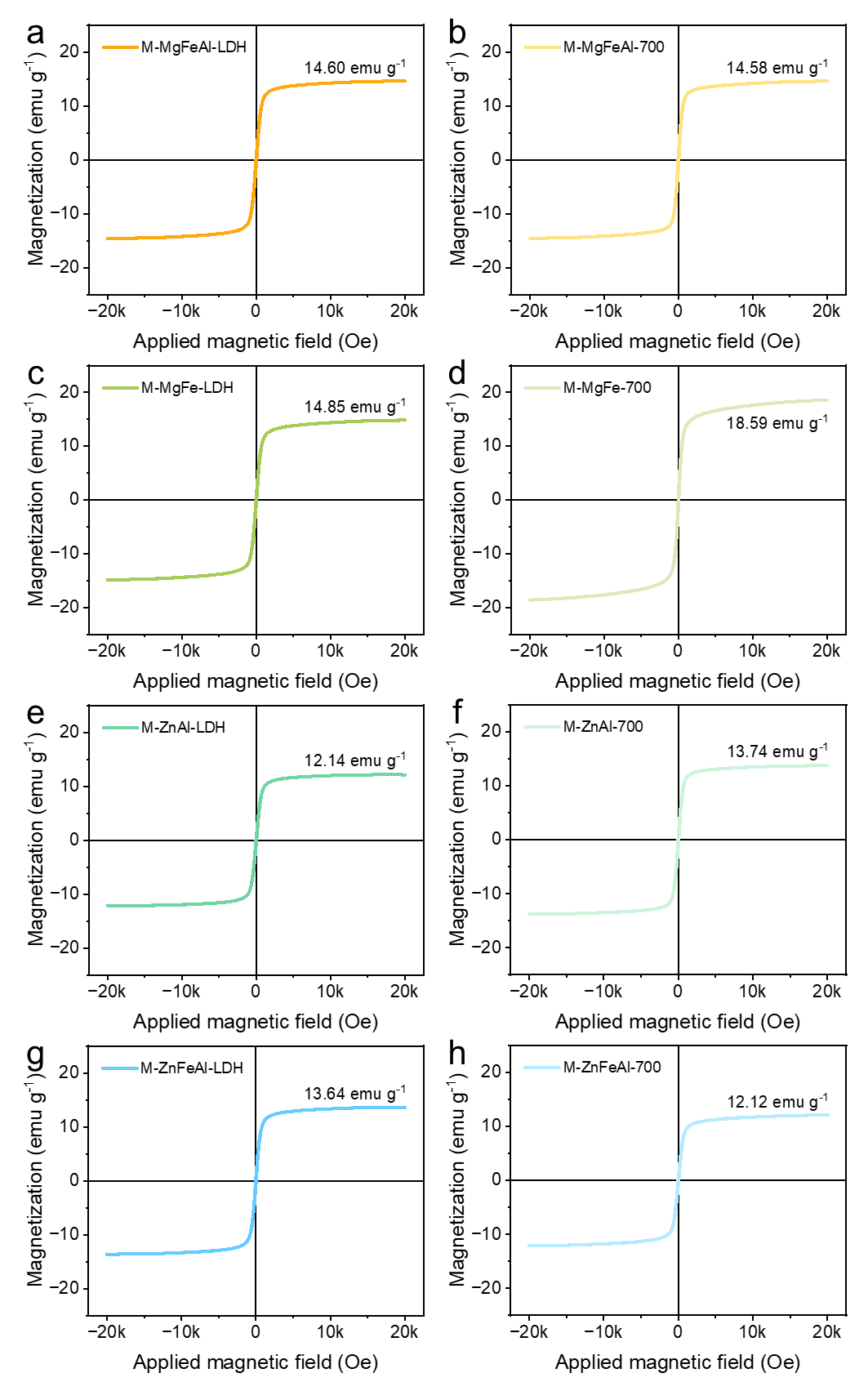


**Figure S15.** Room-temperature magnetization hysteresis loops of the various magnetic LDHs and MMOs.


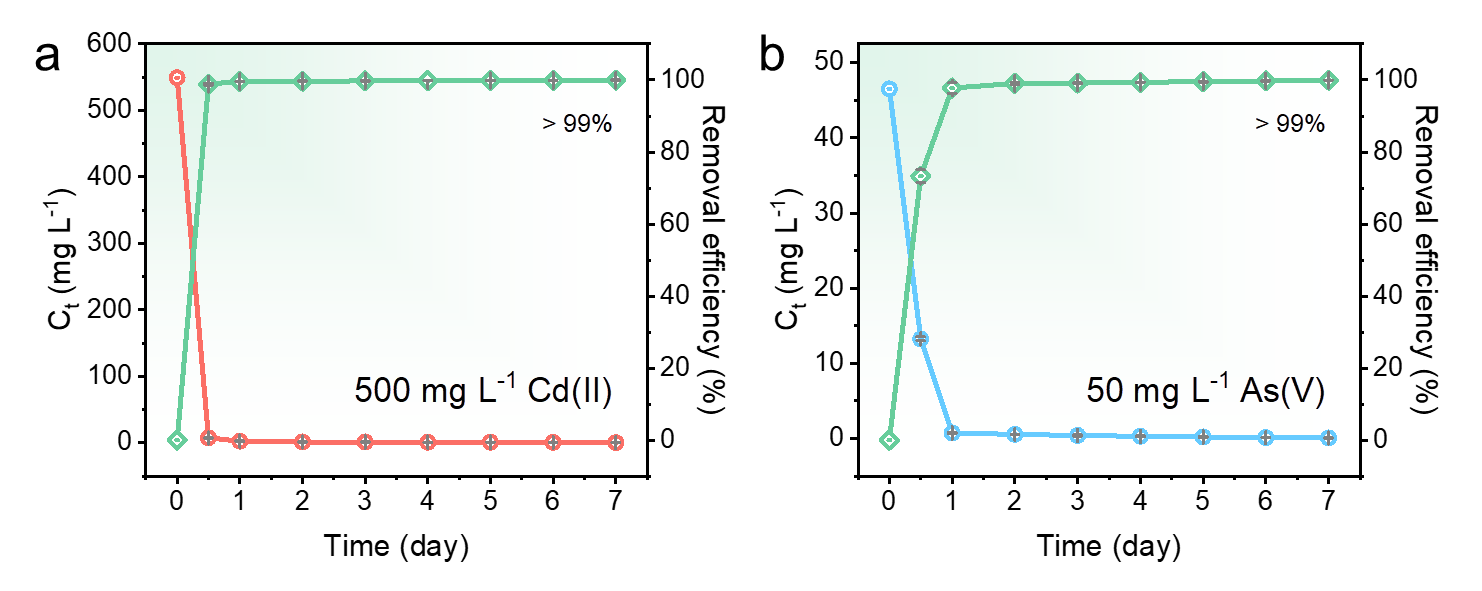


**Figure S16.** C_t_ and removal efficiencies of M-MgAl-700 in (a) Cd(II) and (b) As(V) solution with the different mineralization times.


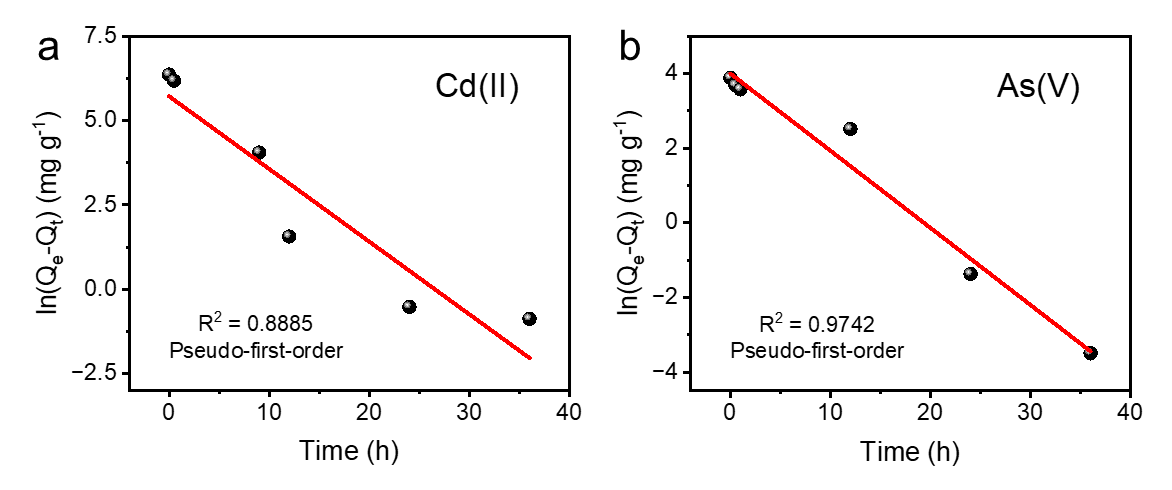


**Figure S17.** Pseudo-first-order adsorption kinetic model fitted by linear regression of M-MgAl-700 for individual (a) Cd(II) and (b) As(V).


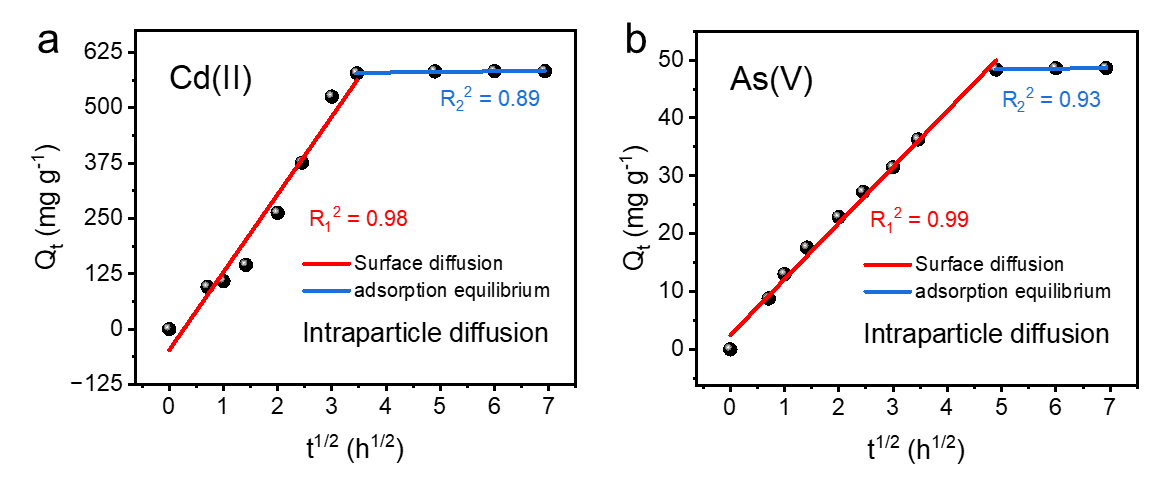


**Figure S18.** Intraparticle diffusion model fitted by linear regression of M-MgAl-700 for individual (a) Cd(II) and (b) As(V).


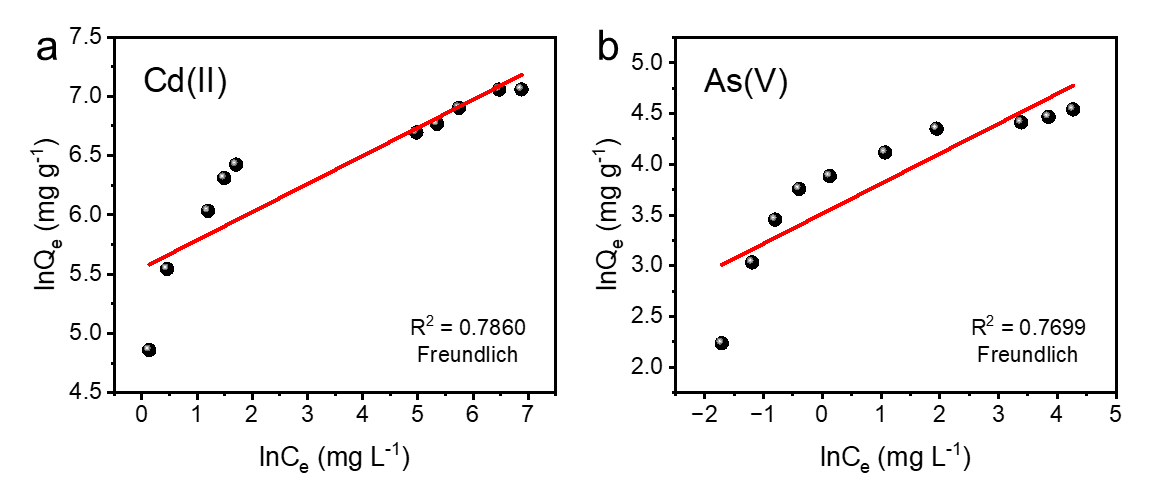


**Figure S19.** Freundlich adsorption isotherms model fitted by linear regression of M-MgAl-700 for individual (a) Cd(II) and (b) As(V).

**Figure S20.** FT-IR spectrum of M-MgAl-700 after Cd(II) mineralization.


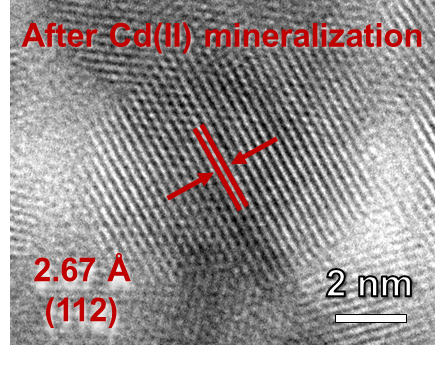


**Figure S21.** HRTEM image of M-MgAl-700 after Cd(II) mineralization.





**Figure S22.** Zeta potential diagram of M-MgAl-700 before and after Cd(II) mineralization.





**Figure S23.** XRD patterns of M-MgAl-700 dispersed in DI in different times.





**Figure S24.** XRD patterns of M-MgAl-700 before and after As(V) mineralization.


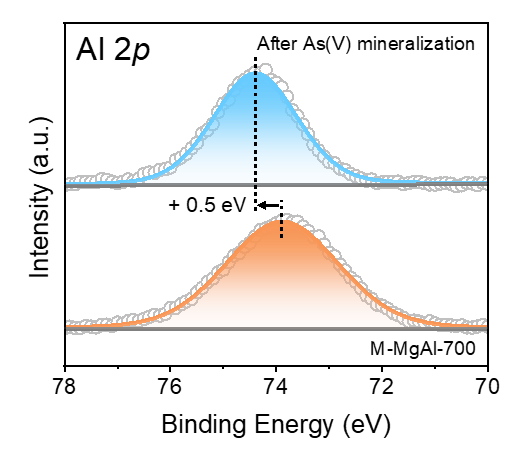


**Figure S25.** Al 2*p* XPS spectra of M-MgAl-700 before and after As(V) mineralization.





**Figure S26.** As K-edge *k*^3^χ (*k*) oscillation spectra for NaAsO_2_, Na_2_HAsO_4_·7H_2_O, and M-MgAl-700 after As(V) mineralization (denoted as MgAl-700-As(V)) and its corresponding *R*-space data.


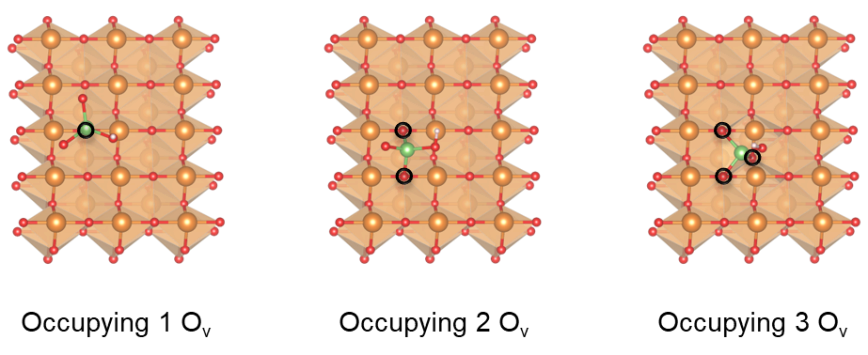


**Figure S27.** Possible modes of M-MgAl-700 after As(V) mineralization from the top view.

**
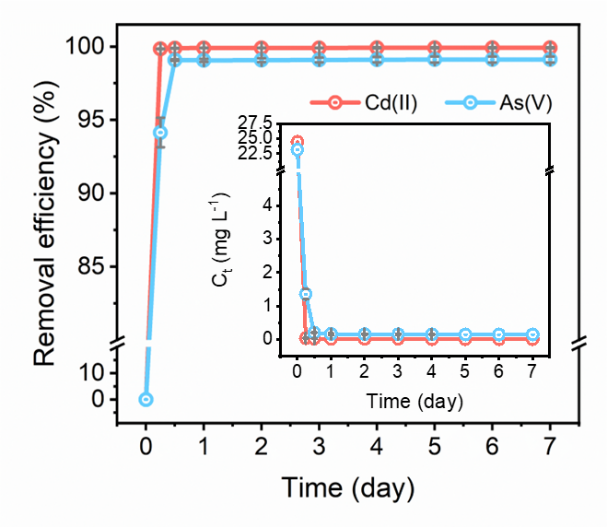
**

**Figure S28.** Removal efficiencies and C_t_ of M-MgAl-700 in coexisting Cd(II) and As(V) solution with the different mineralization times.


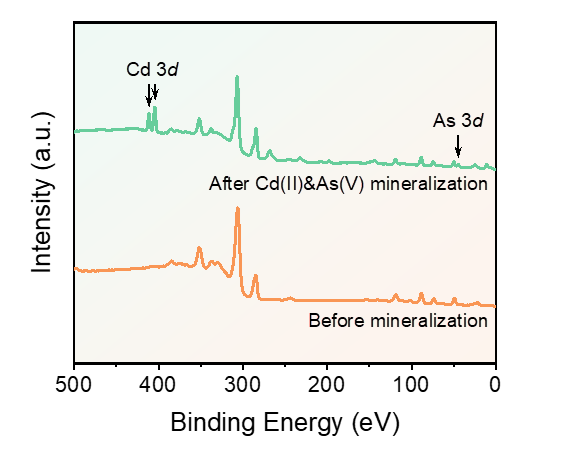


**Figure S29.** XPS survey spectra of M-MgAl-700 before and after Cd(II) and As(V) mineralization.

**
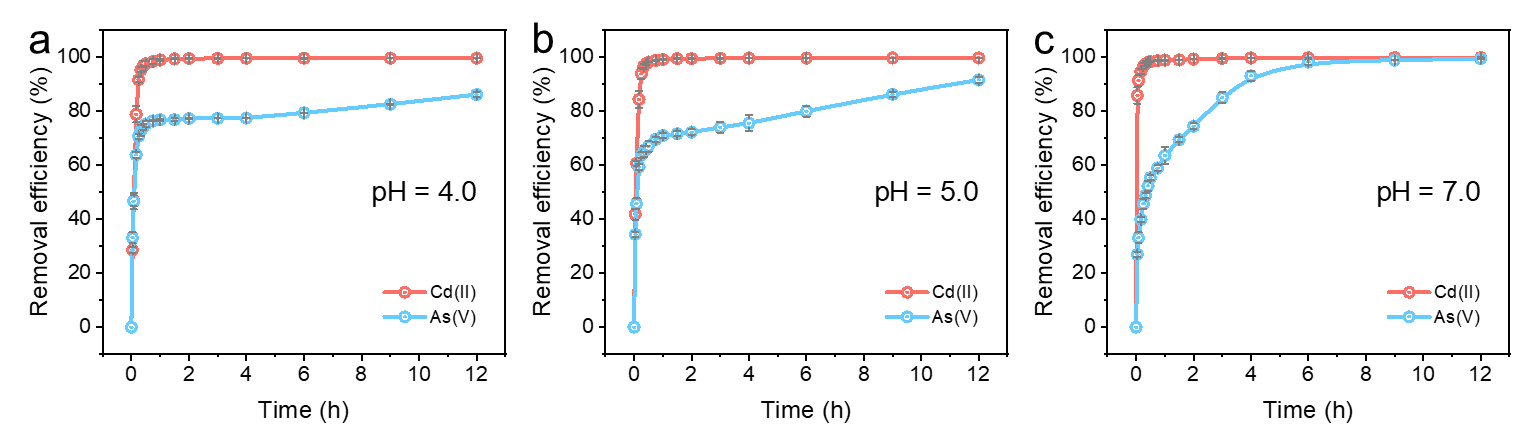
**

**Figure S30.** The simultaneous mineralization experiments using M-MgAl-700 in coexisting Cd(II) and As(V) aqueous solution with initial concentration for each ion of 20 mg L^−1^ and pH value of (a) 4.0, (b) 5.0, and (c) 7.0.

**
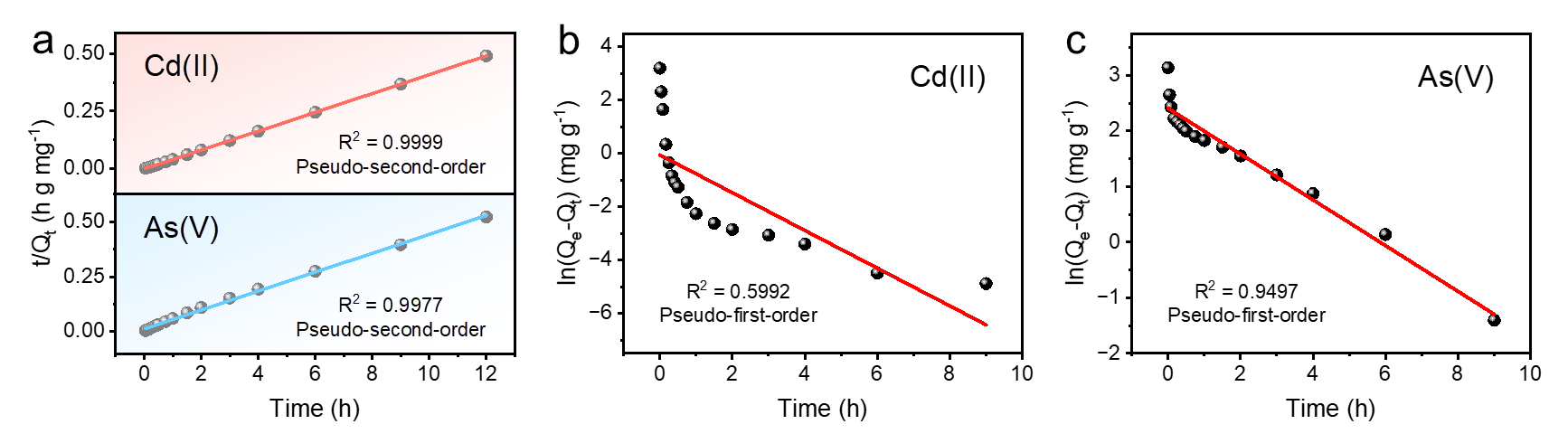
**

**Figure S31.** (a) Pseudo-first-order and (b, c) pseudo-second-order adsorption kinetic models fitted by linear regression of M-MgAl-700 for coexisting Cd(II) and As(V) with initial pH value of 6.0, respectively.





**Figure S32.** Comparison of the removal efficiencies for Cd(II) and As(V) under different coexisting ions.


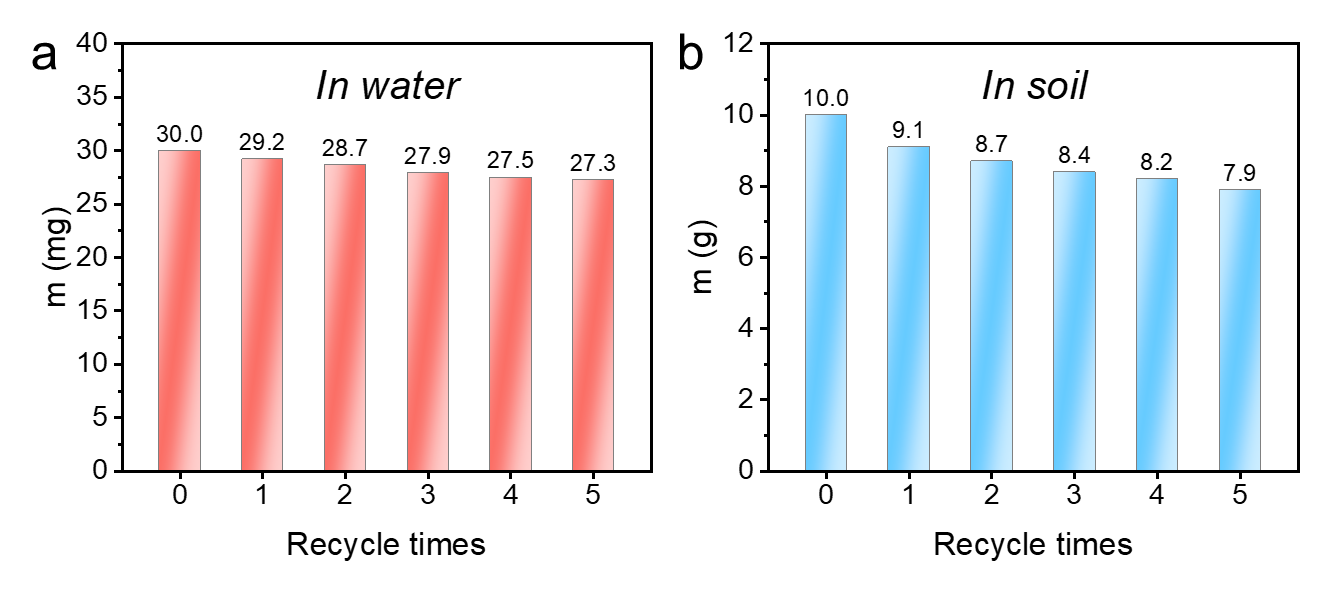


**Figure S33.** The quality of the mineralizer/mineralized product during repeated five-time cycles in (a) water and (b) soil.

**Table S1.** The ion concentration of the solution and atomic ratio after digestion of M-MgAl-700.

|  | **Fe (%)** | **Si (%)** | **Mg (%)** | **Al (%)** |
| --- | --- | --- | --- | --- |
| **Concentration (mg L^‒1^)** | 4.07 | 0.32 | 1.64 | 0.89 |
| **Atomic (%)** | 39.4 | 6.2 | 36.5 | 17.9 |

**Table S2.** The fitting data of pseudo-first-order and pseudo-second-order adsorption kinetic models of M-MgAl-700 for individual Cd(II) and As(V).

| Models | Parameters | Values | |
| --- | --- | --- | --- |
|  |  | **Cd(II)** | **As(V)** |
| Pseudo-first-order (PFO) | k_1_ (h^‒1^) | 0.4970 | 0.4760 |
|  | q_e_ (cal) (mg g^‒1^) | 306.89 | 54.40 |
|  | R^2^ | 0.8885 | 0.9742 |
| Pseudo-second-order (PSO) | k_2_ (g mg^‒1^ h^‒1^) | 1.04·10^−3^ | 6.02·10^−3^ |
|  | q_e_ (cal) (mg g^‒1^) | 609.76 | 52.27 |
|  | R^2^ | **0.9984** | **0.9963** |

**Table S3.** The fitting data of Interparticle diffusion adsorption kinetic models of M-MgAl-700 for individual Cd(II) and As(V).

| Models | Parameters | Values | |
| --- | --- | --- | --- |
|  |  | **Cd(II)** | **As(V)** |
| Interparticle diffusion | k_I1_ (mg g^‒1^ h^‒1/2^) | 175.80 | 9.70 |
|  | C_1_ (mg g^‒1^) | -47.07 | 2.44 |
|  | R_1_^2^ | 0.98 | 0.99 |
|  | k_I2_ (mg g^‒1^ h^‒1/2^) | 1.33 | 0.1274 |
|  | C_2_ (mg g^‒1^) | 574.19 | 47.78 |
|  | R_2_^2^ | 0.89 | 0.93 |

**Table S4.** The fitting data of Langmuir and Freundlich adsorption isotherm models of M-MgAl-700 for individual Cd(II) and As(V).

| Models | Parameters | Values | |
| --- | --- | --- | --- |
|  |  | **Cd(II)** | **As(V)** |
| Langmuir | k_L_ (L mg^‒1^) | 4.14·10^−2^ | 0.6845 |
|  | q_max_ (cal) (mg g^‒1^) | 1173.80 | 93.11 |
|  | R^2^ | **0.9927** | **0.9976** |
| Freundlich | n | 4.2157 | 3.38 |
|  | k_F_ ((mg g^‒1^)(L mg^‒1^)^1/n^) | 257.11 | 33.58 |
|  | R^2^ | 0.7860 | 0.7700 |

**Table S5.** Comparison of removal performance for Cd(II) and As(V) by different mineralizers.

| **Num.** | **Mineralizers** | **Dosage (g L^−1^)** | **T (^o^C)** | **pH value** | | **C_0_ (mg L^−1^)** | | **Q_m_ (mg g^−1^)** | | **Ref.** |
| --- | --- | --- | --- | --- | --- | --- | --- | --- | --- | --- |
|  |  |  |  | **Cd(II)** | **As(V)** | **Cd(II)** | **As(V)** | **Cd(II)** | **As(V)** |  |
| 1 | BCM-1 | 1.0 | 25.0 | 6.1 | 5.9 | 100 | 100 | 111.0 | 60.3 | *Chem. Eng. J.* **2025**, 524, 169442 |
| 2 | FBC | 1.0 | 25.0 | ~7.0 | ~7.0 | 200 | 100 | 129.3 | 32.0 | *Biochar* **2023**, 5, 53 |
| 3 | Fe-Mn nodules | 10.0 | 25.0 | 5.0 | 7.8 | 100 | 4.1 | 129.9 | 9.5 | *J. Hazard. Mater.* **2020**, 390, 122165 |
| 4 | GO-OM | 1.0 | 25.0 | 7.0 | 7.0 | 50.0 | 50.0 | 114.3 | 65.9 | *ACS Appl. Nano Mater.* **2020**, 3, 806 |
| 5 | MgAl-CO_3_ | 1.0 | 25.0 | 6.2 | 8.5 | 1000 | 750 | 237.5 | 30.7 | *Sep. Purif. Technol.* **2025**, 362, 131853 |
| 6 | MgFe-NO_3_ | 1.0 | 25.0 | 5.2 | 5.2 | 1250 | 600 | 387.6 | 92.5 | *Chem. Eur. J.* **2025**, 31, e202403877 |
| 7 | MGO | 1.0 | 25.0 | 5.0 | 5.0 | 100 | 10.0 | 234.0 | 14.0 | *Chem. Eng. J.* **2019**, 358, 1399 |
| 8 | Mn_x_La_1-x_@HTCC | 0.25 | 25.0 | ~6.5 | 4.0 | 50.0 | 20.0 | 64.3 | 39.7 | *Chem. Eng. J.* **2022**, 429, 132262 |
| 9 | NZVI-Coffee ground | 1.0 | 20.0 | 6.0 | 7.0 | ~10.0 | ~10.0 | 112.5 | 9.3 | *Waste Manage.* **2019**, 92, 49 |
| 10 | SBNa800 | 2.0 | 25.0 | ~5.0 | ~3.0 | 50.0 | 50.0 | 109.2 | 59.8 | *J. Hazard. Mater.* **2023**, 447, 130784 |
| 11 | SiO_2_−Al_2_O_3_−Silane-SH | 2.0 | 25.0 | 4.0 | 4.0 | 100 | 100 | 3.7 | 20.4 | *ACS Appl. Mater. Interfaces* **2024**, 16, 34030 |
| 12 | S600 | 1.0 | 25.0 | 4.0 | 4.0 | 1000 | 300 | 98.3 | 64.3 | *Environ. Technol. Innov.* **2024**, 35, 103726 |
| 13 | UiO-66-SH | 0.01 | 25.0 | 5.0 | 5.0 | 10.0 | 10.0 | 77.4 | 52.3 | *Sep. Purif. Technol.* **2024**, 332, 125876 |
| 14 | Z-NZVI | 0.5 | 25.0 | 5.5 | 7.0 | 100 | 10.0 | 62.0 | 12.8 | *J. Hazard. Mater.* **2018**, 344, 1 |
| **15** | **M-MgAl-700** | 1.0 | 25.0 | 6.1 | 8.4 | 2000 | 200 | **1173.8** | **93.1** | **This work** |

**Table S6.** The fitting data of pseudo-first-order and pseudo-second-order adsorption kinetic models of M-MgAl-700 for coexisting Cd(II) and As(V).

| Models | Parameters | Values | |
| --- | --- | --- | --- |
|  |  | **Cd(II)** | **As(V)** |
| Pseudo-first-order (PFO) | k_1_ (h^‒1^) | 1.63 | 0.9475 |
|  | q_e_ (cal) (mg g^‒1^) | 0.9664 | 11.10 |
|  | R^2^ | 0.5992 | 0.9497 |
| Pseudo-second-order (PSO) | k_2_ (g mg^‒1^ h^‒1^) | 3.97 | 0.1530 |
|  | q_e_ (cal) (mg g^‒1^) | 24.53 | 23.21 |
|  | R^2^ | **0.9999** | **0.9977** |

**Table S7.** Theoretical and measured values of the Cd(II) and As(V) concentrations in simulated polluted soils.

| **Location** | **Concentration (mg kg^‒1^)** | **Cd** | **As** |
| --- | --- | --- | --- |
| Yanqing District, Beijing City | Theoretical value | 1.0 | 20.0 |
|  | Measured value | **2.77** | **19.98** |
| Guiyang City, Guizhou Province | Theoretical value | 5.0 | 50.0 |
|  | Measured value | **5.04** | **87.4** |
| Lanxi City, Zhejiang Province | Theoretical value | 0.5 | 10.0 |
|  | Measured value | **1.39** | **16.59** |
| Huangshi City, Hubei Province | Theoretical value | 5.0 | 70.0 |
|  | Measured value | **5.875** | **68.005** |

**Table S8.** Measured values of the contents of common anions in simulated polluted soils.

| **Location** | **Concentration (mg kg^‒1^)** | | | |
| --- | --- | --- | --- | --- |
|  | **PO_4_^3‒^** | **Silicate (SiO_2_, SiO_3_^2‒^)** | **Cl^‒^** | **SO_4_^2‒^** |
| Yanqing District, Beijing City | 1669.65 | 5982.26 | 52.70 | 1425.55 |
| Guiyang City, Guizhou Province | 1809.20 | 11047.18 | 62.25 | 727.30 |
| Lanxi City, Zhejiang Province | 1821.40 | 7179.14 | 171.00 | 570.50 |
| Huangshi City, Hubei Province | 1804.15 | 10492.56 | 70.95 | 1500.60 |
